# The interactions between genetics and early childhood nutrition influence adult cardiometabolic risk factors

**DOI:** 10.1038/s41598-021-94206-4

**Published:** 2021-07-21

**Authors:** Carol A. Wang, John R. Attia, Stephen J. Lye, Wendy H. Oddy, Lawrence Beilin, Trevor A. Mori, Claire Meyerkort, Craig E. Pennell

**Affiliations:** 1 School of Medicine and Public Health, University of Newcastle, Newcastle, NSW Australia; 2 Hunter Medical Research Institute, Newcastle, NSW Australia; 3 Alliance for Human Development, Lunenfeld-Tanenbaum Research Institute, Sinai Health System, Toronto, ON Canada; 4 Menzies Institute for Medical Research, University of Tasmania, Hobart, Australia; 5 Medical School, Royal Perth Hospital Unit, University of Western Australia, Crawley, WA Australia; 6 Sir Charles Gairdner Hospital, Nedlands, WA Australia

**Keywords:** Genetics, Diseases, Endocrinology, Risk factors

## Abstract

It is well established that genetics, environment, and interplay between them play a crucial role in adult disease. We aimed to evaluate the role of genetics, early life nutrition, and the interaction between them, on optimal adult health. As part of a large international consortium (n ~ 154,000), we identified 60 SNPs associated with both birthweight and adult disease. Utilising the Raine Study, we developed a birthweight polygenic score (BW-PGS) based on the 60 SNPs and examined relationships between BW-PGS and adulthood cardiovascular risk factors, specifically evaluating interactions with early life nutrition. Healthy nutrition was beneficial for all individuals; longer duration of any breastfeeding was particularly associated with lower BMI and lower Systolic Blood Pressure in those with higher BW-PGS. Optimal breastfeeding offers the greatest benefit to reduce adult obesity and hypertension in those genetically predisposed to high birthweight. This provides an example of how precision medicine in early life can improve adult health.

## Introduction

Non-communicable diseases (NCDs) represent the largest burden of mortality, accounting for 41 M deaths (71% of annual global mortality) in 2017^1^ with cardiovascular disease (CVD) and diabetes being the two most significant contributors^2^. Geoffrey Rose argued that most cases of disease come from the large proportion of the population at low risk rather than the small proportion at high risk^3^. This has been the foundation of public health campaigns that target the entire population; however, despite decades of public health campaigns, the global rates of some of the major risk factors for CVD (obesity, hypertension, dyslipidaemia and diabetes) continue to increase^1^. It is clear that alternative approaches beyond public health campaigns are required to tackle the global challenge of NCDs. Precision medicine is an approach to medicine where medical decisions and treatments prescribed are tailored in accordance with the recipients’ characteristics as opposed to a one-size-fits-all approach. Over the last decade, it is becoming more evident that precision medicine could potentially be the tool needed to identify those for whom more targeted preventative measures are warranted.

There is substantial evidence that early life exposures program the fetus and infant for lifelong health or disease^4^. This concept, termed the Developmental Origins of Health and Disease (DOHaD) initially focused on birthweight as a surrogate for intrauterine exposures^5,6^; more recent evidence has demonstrated that genetics plays a fundamental role in the relationship between early life events and adult disease^7,8^. Developmental plasticity, the property of a given genotype to produce different phenotypes in response to different environmental conditions, is greatest in the first 1000 days of life^9^. The integration of the key role of genetics in DOHaD and developmental plasticity offers the unique opportunity to utilise precision medicine approaches to reduce NCDs through primary prevention. With this knowledge, enhanced prevention or therapeutic strategies can be particularly targeted to those for whom it will be most efficacious, thus reducing unnecessary treatments and saving costs to the health system^10^.

There is a large body of evidence that adverse antenatal and postnatal environments could program the fetus and infant for lifelong health or disease^4^; however, not all individuals exposed develop poor outcomes as adults suggesting the potential role of genetics in the development of adult disease^7,8^. These studies demonstrate the need to examine the role of genetics, the environment, and the interplay between them to tackle the global challenge of NCDs. We utilised data from the Raine Study and evaluated the associations between genetics, early life nutrition, and adult cardiovascular risk factors, specifically assessing the interaction between genetics and early life nutrition on adult health outcomes.

In this report we provide evidence that the primary prevention of NCDs is possible through DOHaD, developmental plasticity, and precision medicine. The aim of this study was to evaluate the potential for targeted nutritional intervention in early life to reduce the risk of adult disease.

## Results

### Study characteristics

There were 1328 participants available for analyses. Maternal and early life characteristics, and adult measures are summarised by sex in Table 1. On average, the Raine Study male Gen2 participants were heavier and longer at birth and had greater weight gain in the first year compared to their female counterparts. As adults, males were taller, heavier and had higher systolic blood pressure (SBP) than females but lower percent body fat on DEXA scan. Further, males had higher fasting glucose and lower fasting insulin than females. Lipids were similar in both sexes in young adults.

**Table 1:** Demographic table for study participants.

	All Study Participants (N = 1328)	Females (N = 640)	Males (N = 688)
	Mean (SD) or N (%)	Mean (SD) or N (%)	Mean (SD) or N (%)
**Maternal Characteristics**			
Age at pregnancy (in years)	28.80 (5.79)	28.70 (5.84)	28.90 (5.74)
Completed high school N (%)	572 (43.10)	267 (41.78)	305 (44.33)
Nulliparous, N (%)	622 (46.84)	294 (45.94)	328 (47.67)
Height (in m)	1.64 (0.07)	1.64 (0.07)	1.64 (0.06)
Pre-pregnancy weight (in kg)	60.68 (12.19)	60.70 (12.30)	60.70 (12.10)
Pre-pregnancy BMI (in kg/m^2^)	22.53 (4.32)	22.50 (4.28)	22.60 (4.35)
Weight gain in pregnancy as a percentage of pre-pregnancy weight	23.63 (10.51)	23.50 (11.00)	23.70 (10.00)
Diabetes, N (%)	42 (3.21)	18 (2.85)	24 (3.55)
Hypertension, N (%)	336 (25.36)	154 (24.06)	182 (26.57)
Ever smoked during pregnancy, N (%)	463 (34.86)	236 (36.88)	227 (32.99)
**Early Life Characteristics**			
Polygenic Birth Weight Score (BW-PGS)	65.77 (3.78)	65.70 (3.76)	65.80 (3.79)
Gestational age at birth (days)	279 (8.96)	279 (8.81)	279 (9.10)
Birth Length (cm)	49.54 (2.07)	49.20 (1.97)	49.90 (2.10)
Birth Weight (g)	3469.45 (450.83)	3412.00 (442.00)	3523.00 (453.00)
Percent Optimal Birth Weight (POBW)	99.00 (11.61)	99.10 (11.50)	98.90 (11.70)
Duration of any breastfeeding (months)	8.02 (7.14)	7.92 (7.19)	8.11 (7.09)
Duration of exclusive breastfeeding (months)	3.13 (1.95)	3.09 (1.97)	3.17 (1.94)
Weight gain in the first year of life as a percentage of birth weight	202.74 (43.69)	197.00 (43.70)	208.00 (43.20)
**Characteristics at age 20**			
Percentage Body Fat	30.25 (12.77)	39.60 (9.01)	21.60 (9.08)
Bone Mass Density	1.08 (0.11)	1.03 (0.09)	1.13 (0.11)
**Characteristics at age 22**			
Currently smoking, N (%)	118 (15.49)	55 (13.82)	63 (17.31)
Height (in m)	1.73 (0.09)	1.66 (0.06)	1.80 (0.07)
Weight (in kg)	76.37 (17.53)	70.90 (17.70)	81.70 (15.60)
BMI (in kg/m^2^)	25.24 (5.15)	25.20 (5.82)	25.20 (4.43)
Waist (in cm)	83.68 (13.75)	81.20 (15.20)	86.10 (11.80)
Hip (in cm)	100.36 (11.21)	101.00 (12.80)	100.00 (9.41)
Waist-Hip ratio	0.83 (0.08)	0.80 (0.08)	0.86 (0.06)
Systolic BP (in mmHg)	119 (11.22)	114 (9.77)	123 (10.80)
Diastolic BP (in mmHg)	67 (7.07)	67.30 (7.00)	66.70 (7.14)
Glucose_F_ (in mmol/l)	5.02 (0.85)	4.86 (0.40)	5.17 (1.11)
Insulin_F_ (in mU/ml)	8.39 (5.48)	9.31 (6.25)	7.52 (4.46)
HOMA-IR_F_	1.89 (1.37)	2.06 (1.59)	1.73 (1.10)
Total Cholesterol_F_ (in mmol/l)	4.61 (0.83)	4.71 (0.83)	4.50 (0.82)
Triglycerides_F_ (in mmol/l)	1.09 (0.48)	1.07 (0.45)	1.11 (0.51)
HDL-Cholesterol_F_ (in mmol/l)	1.36 (0.34)	1.48 (0.39)	1.24 (0.24)
LDL-Cholesterol_F_ (in mmol/l)	2.74 (0.71)	2.74 (0.67)	2.75 (0.75)

F denotes fasting.

### The development of a birthweight polygenic score

The use of polygenic scores (PGS) has been shown to provide more power with less bias than single SNP approaches. These scores have an expanding role in predicting disease and can potentially enable a better understanding of aetiology of disease. In addition, PGS have also been used to test for genome-wide Gene–Gene and Gene-Environment interactions^11,12^. As part of a meta-analysis within a large international consortium (n ~ 154,000), we identified 60 SNPs associated with birthweight^7^ (Supplementary Table 1) and developed a birthweight PGS (BW-PGS; see Methods) for each study participant. The BW-PGS was then used as a genetic measure in the evaluation of associations between genetics, early life nutrition, and adult cardiovascular risk factors.

### Breastfeeding in individuals who are genetically predisposed to high birthweight reduces the risk of obesity

**Figure 1:**
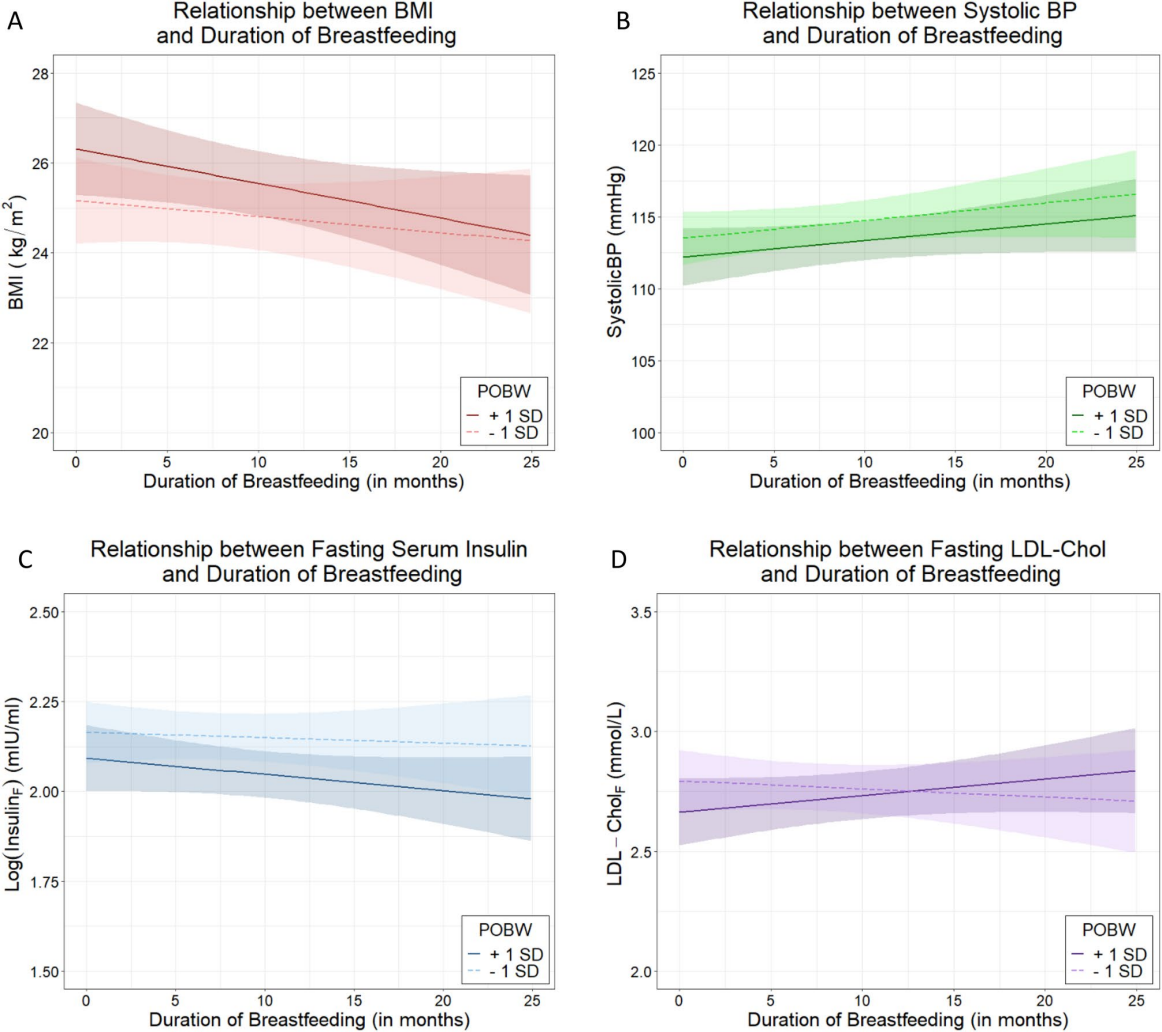
The relationship between duration of any breastfeeding and health measures for POBW that is one standard deviation above the mean, and for POBW that is one standard deviation below the mean. F denotes fasting. (**A**): Body mass index (BMI) at 22 years of age; (**B**): Systolic blood pressure (SBP) at 22 years of age; (**C**): Fasting insulin levels at 22 years of age; and (**D**): Fasting low density lipoprotein cholesterol (LDL-c) at 22 years of age (Figure generated using ggplot2 in Refs.^37,38^).

There was no association between percent optimal birthweight (POBW) and body mass index (BMI) at age 22 (p = 0.063, Table 2). The duration of any breastfeeding and Eating Assessment in Toddlers score at 1 year (EAT_1_) were associated with reduced adult BMI (p = 0.050 and p = 0.010, respectively). There was no evidence of effect modification of breastfeeding on BMI by POBW (p = 0.43, Table 2, Fig. 1A, Supplementary Fig. 1A).

**Table 2:** Association analyses for body mass index (kg/m^2^).

Percent Optimal Birth Weight (POBW)	Model 1^α^		Model 2^β^	
Predictors	Estimate (95% CI)	P	Estimate (95% CI)	P
Intercept	27.62 (25.69–29.55)	** < 0.0001**	27.70 (25.76–29.65)	** < 0.0001**
POBW^γ^	0.40 (−0.02 to 0.82)	0.063	0.41 (−0.01 to 0.83)	0.056
Duration BF (months)^†^	−0.06 (−0.12 to 0.00)	**0.050**	−0.06 (−0.12 to 0.00)	0.064
Sex (M)	0.16 (−0.66 to 0.98)	0.70	0.12 (−0.71 to 0.95)	0.78
EAT_1_ score^‡^	−0.06 (−0.10 to −0.01)	**0.010**	−0.06 (−0.10 to −0.01)	**0.0091**
POBW * Duration BF			−0.02 (−0.07 to 0.03)	0.43

Birthweight Polygenic Score (BW-PGS)	Model 3^ε^		Model 4^ϕ^	
Predictors	Estimate (95% CI)	P	Estimate (95% CI)	P
Intercept	27.67 (25.73–29.62)	** < 0.0001**	27.62 (25.68–29.55)	** < 0.0001**
BW-PGS^§^	0.10 (−0.33 to 0.53)	0.64	0.18 (−0.25 to 0.61)	0.058
Duration BF (months)^†^	−0.05 (−0.11 to 0.01)	0.10	−0.05 (−0.11 to 0.01)	0.11
Sex (M)	0.19 (−0.63 to 1.02)	0.64	0.24 (−0.58 to 1.06)	0.57
EAT_1_ score^‡^	−0.06 (−0.10 to −0.02)	**0.0084**	−0.06 (−0.10 to −0.01)	**0.0090**
BW-PGS * Duration BF			−0.06 (−0.11 to −0.00)	**0.037**

^γ^POBW = Percent Optimal Birth Weight (standardised); ^†^ Duration of BF = Duration of any breastfeeding (mean-centred); ^‡^ EAT_1_ score = quality of early life nutrition in first year of life; ^§^ BW-PGS = birth weight polygenic score (standardised); ^δ^ adjustment for population stratification; ^α^ Model 1 examines the effect of duration of breastfeeding adjusting for POBW, sex and EAT_1_ score; ^β^ Model 2 examines the effect modification of duration of breastfeeding by POBW adjusting for sex and EAT_1_ score; ^ε^ Model 3 examines the effect of duration of breastfeeding adjusting for BW-PGS, sex, EAT_1_ score and the first two principal components [PCs] (estimates not presented); ^ϕ^ Model 4 examines the effect modification of duration of breastfeeding by BW-PGS adjusting for sex, EAT_1_ score and the first two principal components [PCs] (estimates not presented).

**Figure 2:**
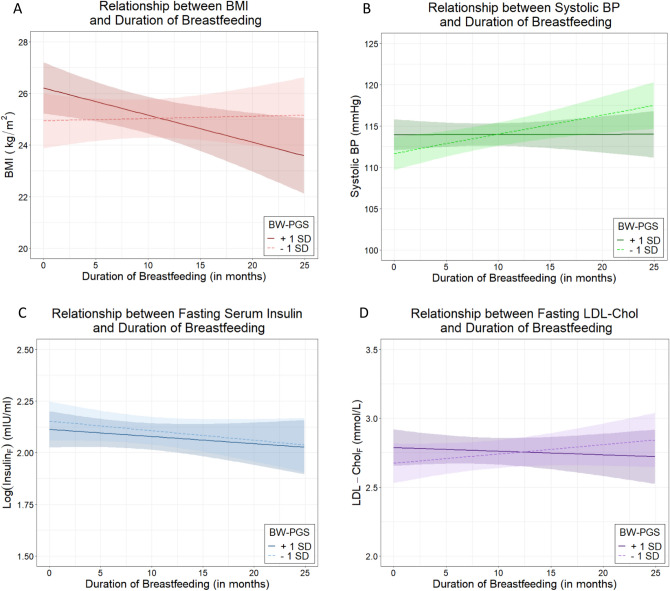
The relationship between duration of any breastfeeding and health measures for BW-PGS that is one standard deviation above the mean, and for BW-PGS that is one standard deviation below the mean. F denotes fasting. (**A**): Body mass index (BMI) at 22 years of age; (**B**): Systolic blood pressure (SBP) at 22 years of age; (**C**): Fasting insulin levels at 22 years of age; and (**D**): Fasting low density lipoprotein cholesterol (LDL-c) at 22 years of age (Figure generated using ggplot2 in Refs.^37,38^).

Similar to POBW, no association was observed between BW-PGS and BMI (p = 0.64. Table 2). Healthy early nutrition beyond breastfeeding at one year of age (EAT_1_ score; p = 0.0084, Table 2) and three years of age (EAT_3_ score; p = 0.043, Supplementary Table 2) were associated with lower BMI. There was an interaction between breastfeeding and BW-PGS on BMI, such that lower BMI was observed in those that were breastfed longer and had higher BW-PGS (p = 0.037, Table 2, Fig. 2A, Supplementary Fig. 1B).

**Figure 3:**
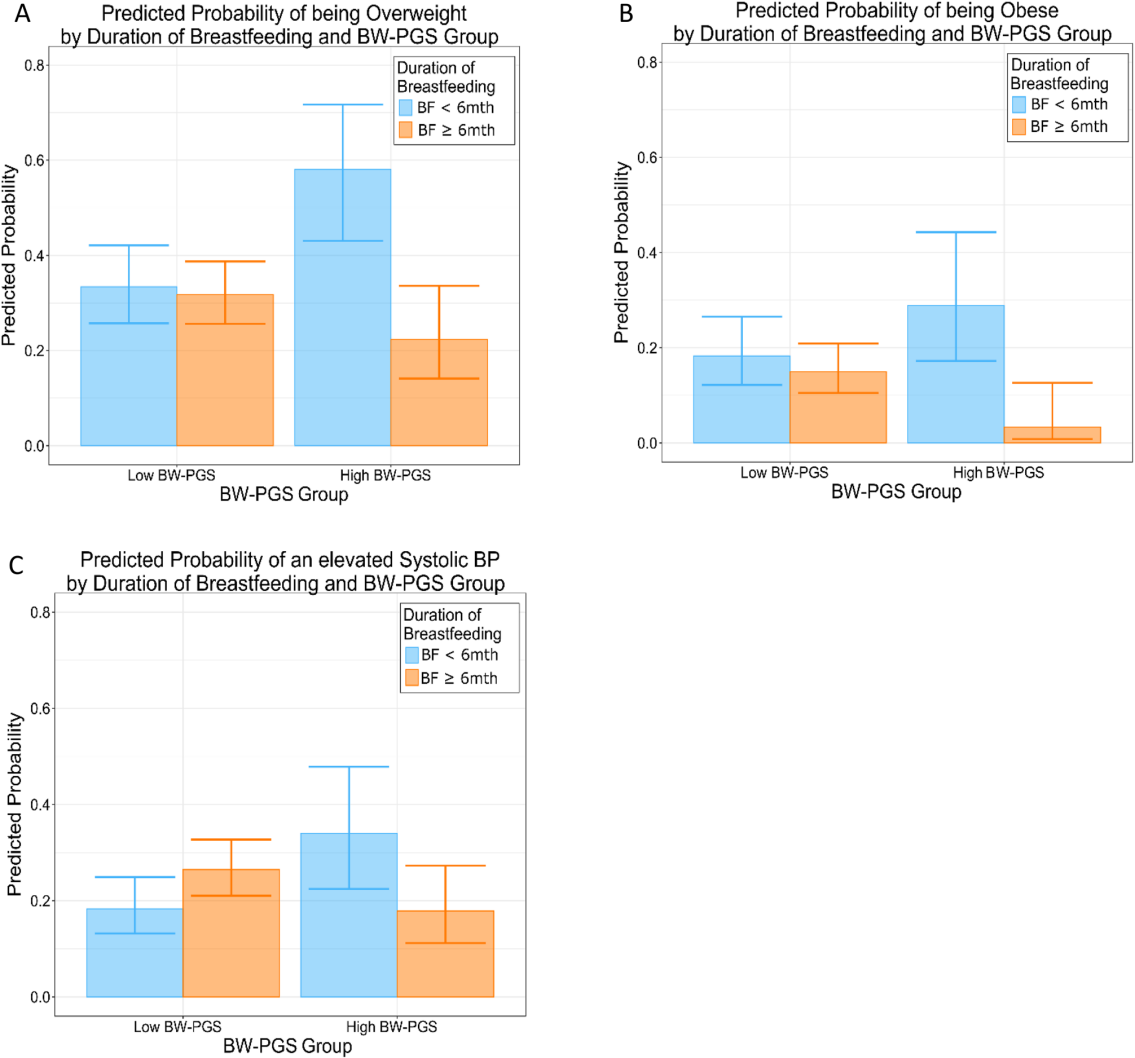
Predicted probability of adverse adult health outcomes at 22 years of age by BW-PGS group (High vs Low) and Duration of any Breastfeeding (< 6 months vs ≥ 6 months). A low BW-PGS is defined as those in the 0-80th percentile of the BW-PGS within the Raine Study participants, while a high BW-PGS is defined as those in the highest quintile (80-100th percentile) of the BW-PGS within the Raine Study participants. (**A**): Predicted probability of being obese (BMI ≥ 25 kg/m^2^) at 22 years of age by BW-PGS and duration of breastfeeding; (**B**): Predicted probability of being obese (BMI ≥ 30 kg/m^2^) at 22 years of age by BW-PGS and duration of breastfeeding; (**C**): Predicted probability of being an elevated systolic BP (SBP ≥ 120 mmHg) at 22 years of age by BW-PGS and duration of breastfeeding. (Figure generated using ggplot2 in Refs.^37,38^).

When obesity was trichotomised using clinical definitions of overweight (BMI ≥ 25 kg/m^2^) and obese (BMI ≥ 30 kg/m^2^), the effect modification of breastfeeding on the outcome by BW-PGS persisted (breastfeeding*BW-PGS interaction p-values p = 0.00096 and p = 0.0079 respectively for overweight and obese outcomes; Supplementary Table 3, Fig. 3A and B).

With early life nutrition beyond breastfeeding, the associations between EAT_1_ score and clinical definitions of overweight and obese persisted while associations between EAT_3_ score and clinical definitions of overweight and obese were no longer significant (EAT_1_ p = 0.024; EAT_3_ p = 0.066; Supplementary Table 4).

Somewhat similar results were observed for association analyses for percent body fat measured by DEXA scan for healthy early life nutrition (β_EAT1 score_ = −0.09, p = 0.0054; Supplementary Table 5) and breastfeeding (β_breastfeeding_ = −0.09, p = 0.054; Supplementary Table 5). Furthermore, there was a suggestive association for effect modification of breastfeeding by BW-PGS (p = 0.069; Supplementary Table 5; Supplementary Figs. 2A and 3A).

As part of sensitivity analyses, we examined the imputed dataset and demonstrated the effect modification of breastfeeding on BMI by BW-PGS (p = 0.038, Supplementary Table 6) was consistent to that of complete case analysis.

### Breastfeeding in individuals who are genetically predisposed to high birthweight reduces the risk of elevated blood pressure

There was no association between POBW and SBP or BW-PGS and SBP. Longer periods of breastfeeding were associated with higher SBP in young adults (p = 0.041, Table 3). Healthy nutrition beyond breastfeeding at one year of age, EAT_1_ score, was associated with lower SBP (Table 3). No association was observed with SBP and improved EAT_3_ score (p = 0.51, Supplementary Table 7). There was no effect modification for breastfeeding on SBP by POBW (p = 0.95, Table 3, Fig. 1B). In contrast, there was an interaction between breastfeeding duration and BW-PGS on SBP (p = 0.030, Table 3, Fig. 2B, Supplementary Fig. 1D), such that higher SBP was observed in those with a low BW-PGS and who were breastfed for shorter duration. This result, however, was not replicated in sensitivity analyses when we evaluated the effect modification of breastfeeding on SBP by BW-PGS (p = 0.15, Supplementary Table 8) in the imputed dataset and was likely due to the small effect size of breastfeeding on SBP. When examining SBP as a dichotomous outcome (SBP ≥ 120 mmHg), those with high BW-PGS who were breastfed for more than six months had lower SBP than those with shorter periods of breastfeeding and similar BW-PGS (p = 0.0022, Supplementary Table 9, Fig. 3C). EAT_1_ score, but not EAT_3_ score, was associated with a lower risk of an SBP ≥ 120 mmHg (EAT_1_ p = 0.022; EAT_3_ p = 0.47; Supplementary Table 10).

**Table 3:** Association analyses for systolic blood pressure (mmHg).

Percent Optimal Birth Weight (POBW)	Model 1^α^		Model 2^β^	
Predictors	Estimate (95% CI)	P	Estimate (95% CI)	P
Intercept	118.15 (114.46–121.83)	** < 0.0001**	118.16 (114.46–121.86)	** < 0.0001**
POBW^γ^	−0.69 (−1.50 to 0.12)	0.096	−0.68 (−1.50 to 0.13)	0.099
Duration BF (months)^†^	0.12 (0.01–0.23)	**0.038**	0.12 (0.01–0.23)	**0.039**
Sex (M)	9.50 (7.93–11.08)	** < 0.0001**	9.50 (7.91–11.09)	** < 0.0001**
EAT_1_ score^‡^	−0.10 (−0.18 to −0.02)	**0.015**	−0.10 (−0.18 to −0.02)	**0.015**
POBW * Duration BF			−0.00 (−0.10 to 0.1)	0.95

Birthweight Polygenic Score (BW-PGS)	Model 3^ε^		Model 4^ϕ^	
Predictors	Estimate (95% CI)	P	Estimate (95% CI)	P
Intercept	118.17 (114.47–121.87)	** < 0.0001**	117.93 (114.23–121.62)	** < 0.0001**
BW-PGS^§^	0.07 (−0.75 to 0.88)	0.87	0.20 (−0.62 to 1.02)	0.63
Duration BF (months)^†^	0.11 (0.00–0.23)	**0.048**	0.12 (0.01–0.23)	**0.041**
Sex (M)	9.49 (7.91–11.06)	** < 0.0001**	9.59 (8.01–11.16)	** < 0.0001**
EAT_1_ score^‡^	−0.10 (−0.18 to −0.02)	**0.015**	−0.10 (−0.18 to −0.02)	**0.020**
BW-PGS * Duration BF			−0.12 (−0.22 to −0.01)	**0.030**

^γ^POBW = Percent Optimal Birth Weight (standardised); ^†^ Duration of BF = Duration of any breastfeeding (mean-centred); ^‡^ EAT_1_ score = quality of early life nutrition in first year of life; ^§^ BW-PGS = birth weight polygenic score (standardised); ^δ^ adjustment for population stratification; ^α^ Model 1 examines the effect of duration of breastfeeding adjusting for POBW, sex and EAT_1_ score; ^β^ Model 2 examines the effect modification of duration of breastfeeding by POBW adjusting for sex and EAT_1_ score; ^ε^ Model 3 examines the effect of duration of breastfeeding adjusting for BW-PGS, sex, EAT_1_ score and the first two principal components [PCs] (estimates not presented); ^ϕ^ Model 4 examines the effect modification of duration of breastfeeding by BW-PGS adjusting for sex, EAT_1_ score and the first two principal components [PCs] (estimates not presented).

There were no associations between DBP and POBW or DBP and BW-PGS. Some associations were demonstrated between duration of breastfeeding and EAT_1_ score with DBP (Supplementary Table 11). No effect modification was demonstrated for breastfeeding on DBP by POBW (p = 0.30) or BW-PGS (p = 0.25).

### Healthy nutrition beyond breastfeeding in early life reduces the risk of diabetes

Fasting serum insulin was inversely associated with POBW and EAT_1_ score; no associations were demonstrated with BW-PGS or breastfeeding or EAT_3_ score (Table 4, Supplementary Table 12). Furthermore, no effect modification was demonstrated for breastfeeding on fasting insulin by POBW or BW-PGS (p = 0.51 and p = 0.81 respectively, Table 4, Figs. 1C and 2C). Paradoxically, data on glucose and HOMA-IR (Supplementary Tables 13 and 14 and Supplementary Figs. 2C, 2D, 3C and 3D) showed a main effect of POBW but not BW-PGS; however, there was no effect modification for breastfeeding on these outcomes by either POBW or BW-PGS.

**Table 4:** Association analyses for Fasting Serum Insulin (Natural Log Transformed).

Percent Optimal Birth Weight (POBW)	Model 1^α^		Model 2^β^	
Predictors	Estimate (95% CI)	P	Estimate (95% CI)	P
Intercept	2.28 (2.11–2.45)	** < 0.0001**	2.29 (2.11–2.46)	** < 0.0001**
POBW^γ^	−0.05 (−0.09 to −0.01)	**0.011**	−0.05 (−0.09 to −0.01)	**0.013**
Duration BF (months)^†^	−0.00 (−0.01 to 0.00)	0.23	−0.00 (−0.01 to 0.00)	0.26
Sex (M)	−0.19 (−0.26 to −0.12)	** < 0.0001**	−0.19 (−0.27 to −0.12)	** < 0.0001**
EAT_1_ score^‡^	−0.00 (−0.01 to −0.00)	**0.032**	−0.00 (−0.01 to −0.00)	**0.030**
POBW * Duration BF			−0.00 (−0.01 to 0.00)	0.51

Birthweight Polygenic Score (BW-PGS)	Model 3^ε^		Model 4^ϕ^	
Predictors	Estimate (95% CI)	P	Estimate (95% CI)	P
Intercept	2.23 (2.08–2.43)	** < 0.0001**	2.26 (2.08–2.43)	** < 0.0001**
BW-PGS^§^	−0.02 (−0.05 to 0.02)	0.45	−0.02 (−0.05 to 0.02)	0.43
Duration BF (months)^†^	−0.00 (−0.01 to 0.00)	0.13	−0.00 (−0.01 to 0.00)	0.13
Sex (M)	−0.19 (−0.26 to −0.11)	** < 0.0001**	−0.19 (−0.26 to −0.11)	** < 0.0001**
EAT_1_ score^‡^	−0.00 (−0.01 to 0.00)	0.067	−0.00 (−0.01 to 0.00)	0.066
BW-PGS * Duration BF			0.00 (−0.00 to 0.01)	0.81

^γ^POBW = Percent Optimal Birth Weight (standardised); ^†^ Duration of BF = Duration of any breastfeeding (mean-centred); ^‡^ EAT_1_ score = quality of early life nutrition in first year of life; ^§^ BW-PGS = birth weight polygenic score (standardised); ^δ^ adjustment for population stratification; ^α^ Model 1 examines the effect of duration of breastfeeding adjusting for POBW, sex and EAT_1_ score; ^β^ Model 2 examines the effect modification of duration of breastfeeding by POBW adjusting for sex and EAT_1_ score; ^ε^ Model 3 examines the effect of duration of breastfeeding adjusting for BW-PGS, sex, EAT_1_ score and the first two principal components [PCs] (estimates not presented); ^ϕ^ Model 4 examines the effect modification of duration of breastfeeding by BW-PGS adjusting for sex, EAT_1_ score and the first two principal components [PCs] (estimates not presented).

### Healthy nutrition beyond breastfeeding in early life reduces the risk of dyslipidaemia

LDL-C was not associated with POBW, BW-PGS, breastfeeding or EAT_3_ score (Table 5, Figs. 1D and 2D, Supplementary Table 15); inverse associations were observed between LDL-C and EAT_1_ score (Table 5). Data for total cholesterol, triglycerides and HDL-C (Supplementary Tables 16–18) showed no effect modification for breastfeeding on any of these lipid measure by POBW or BW-PGS (Supplementary Figs. 2E-G and 3E-G).

**Table 5:** Association analyses for Fasting Low-Density-Lipoprotein-Cholesterol (mmol/L).

Percent Optimal Birth Weight (POBW)	Model 1^α^		Model 2^β^	
Predictors	Estimate (95% CI)	P	Estimate (95% CI)	P
Intercept	3.15 (2.89–3.42)	** < 0.0001**	3.13 (2.87–3.40)	** < 0.0001**
POBW^γ^	−0.02 (−0.08 to 0.04)	0.52	−0.02 (−0.08 to 0.03)	0.43
Duration BF (months)^†^	0.00 (−0.01 to 0.01)	0.56	0.00 (−0.01 to 0.01)	0.66
Sex (M)	0.02 (−0.10 to 0.13)	0.79	0.03 (−0.08 to 0.14)	0.62
EAT_1_ score^‡^	−0.01 (−0.02 to −0.00)	**0.0017**	−0.01 (−0.02 to −0.00)	**0.0022**
POBW * Duration BF			0.01 (−0.00 to 0.01)	0.14

Birthweight Polygenic Score (BW-PGS)	Model 3^ε^		Model 4^ϕ^	
Predictors	Estimate (95% CI)	P	Estimate (95% CI)	P
Intercept	3.15 (2.88–3.41)	** < 0.0001**	3.13 (2.87–3.40)	** < 0.0001**
BW-PGS^§^	0.01 (−0.04 to 0.07)	0.65	0.02 (−0.04 to 0.08)	0.52
Duration BF (months)^†^	0.00 (−0.01 to 0.01)	0.63	0.00 (−0.01 to 0.01)	0.61
Sex (M)	0.02 (−0.10 to 0.13)	0.76	0.02 (−0.09 to 0.13)	0.71
EAT_1_ score^‡^	−0.01 (−0.02 to −0.00)	**0.0019**	−0.01 (−0.02 to −0.00)	**0.0024**
BW-PGS * Duration BF			−0.01 (−0.01 to 0.00)	0.20

^γ^POBW = Percent Optimal Birth Weight (standardised); ^†^ Duration of BF = Duration of any breastfeeding (mean-centred); ^‡^ EAT_1_ score = quality of early life nutrition in first year of life; ^§^ BW-PGS = birth weight polygenic score (standardised); ^δ^ adjustment for population stratification; ^α^ Model 1 examines the effect of duration of breastfeeding adjusting for POBW, sex and EAT_1_ score; ^β^ Model 2 examines the effect modification of duration of breastfeeding by POBW adjusting for sex and EAT_1_ score; ^ε^ Model 3 examines the effect of duration of breastfeeding adjusting for BW-PGS, sex, EAT_1_ score and the first two principal components [PCs] (estimates not presented); ^ϕ^ Model 4 examines the effect modification of duration of breastfeeding by BW–PGS adjusting for sex, EAT_1_ score and the first two principal components [PCs] (estimates not presented).

### There was no evidence of differential effects of healthy nutrition by sex

No differential effect of breastfeeding by sex and BW-PGS was found in three-way interaction analyses for BMI and SBP (Supplementary Tables 19–22); neither was there a significant association when the modifying effect of breastfeeding on BMI and SBP, via sex, was examined in the two-way interactions for sex and breastfeeding (Supplementary Tables 23–24).

## Discussion

In this study, we demonstrated the critical role and potential for nutritional intervention in the first year of life to reduce cardiovascular risk factors, as surrogates for the components of adult metabolic syndrome. Specifically, our results suggested an interaction between duration of any breastfeeding and genetics; greater benefit for breastfeeding was present in the subgroup with increased BW-PGS. Individuals with high BW-PGS had a greater reduction in the incidence of overweight and obesity and lower systolic blood pressure with increased duration of any breastfeeding than those with low BW-PGS. There was a 25% and 35% reduction in adult overweight and obesity, respectively, in those with high BW-PGS with increased duration of any breastfeeding. In contrast, there was no significant difference in those with low BW-PGS with increased duration of any breastfeeding. Similarly, the probability of having elevated systolic blood pressure (≥ 120 mmHg) was approximately halved in those with high BW-PGS with increased duration of any breastfeeding, whereas there was no significant difference in those with low BW-PGS with increased duration of any breastfeeding. While encouraging breastfeeding in all mothers is a good public health strategy, our results suggest that precision medicine, in the form of actively supporting longer duration of any breastfeeding in the subgroup at highest genetic risk, has the potential to place individuals on trajectories to health rather than disease. While it appears the differential effect of breastfeeding by BW-PGS is only present in risk of obesity and elevated blood pressure and not present in insulin and LDL-c, we propose that one possible explanation is that the genetic influence is stronger for BMI and blood pressure, while postnatal environment influences are perhaps greater for insulin and LDL-c.

Next, we demonstrated a beneficial effect of healthy nutrition beyond breastfeeding, with associations more likely to be significant in the earlier years of life rather than later. This is shown through the 20–25% reduction in the probability of adult overweight/obesity with every standard deviation increase in dietary quality score at one year of age (EAT_1_ score), and no association between the risk of overweight/obesity and dietary quality score at three years of age (EAT_3_ score). Similarly, there is an approximate 20% reduction in the risk of SBP ≥ 120 mmHg with every standard deviation increase in EAT_1_ score and no association between SBP and EAT_3_ score. This reinforces the critical importance of nutrition beyond breastfeeding in the first year of life.

Evidence over the last three decades has established an association between adverse intrauterine environment (low birthweight) and adult disease. This relationship was first noted by David Barker and has been successfully replicated in numerous studies^13^. Despite this body of evidence, risk with low birthweight was not demonstrated in our study. The failure to replicate these findings is possibly due to the relatively young age of the cohort and limited variance within the cardiovascular risk factors within our study participants.

The Early Growth Genetics (EGG) consortium recently released a GWAS of birthweight^8^ with an increased number of SNPs identified. The proportion of variance explained in birthweight by the updated genetics score based on these SNPs were, however, half of that of which explained by the BW-GRS used in this study. Considering a greater proportion of variance explained in birthweight weight by BW-GRS used in this study, we are confident that the results presented in this study potentially provides improved statistical power, and is a better instrumental variable compared to the SNPs in the recent 2019 GWAS paper.

In the recent years, it has been suggested that increased disease risk could also be present in babies born large-for-gestational-age^13^. A recent meta-analysis by Knop et. al. replicated the original inverse association with blood pressure but suggested a ‘J-shaped’ relationship with the risk of developing diabetes mellitus and cardiovascular disease^14^. Further, two separate meta-analyses have reported the highest risk of overweight and obesity among large-for-gestational-age babies^5,6^. A growing body of evidence suggests DOHaD is largely driven by genetics^7,8^. As part of two large consortium studies involving more than 100 K individuals from across 35 studies, we demonstrated genetic variants associated with birthweight were also associated with BMI, SBP and risk for later life adult metabolic disease^7,8^, suggesting birthweight could potentially be a surrogate for genetic variations associated with adult disease.

Over the last quarter century, emphasis has been placed upon an individual’s environmental exposure before two years of age. The end of the second year of life marks the end of the first 1000 days since conception, and is a crucial period where environmental exposures could have the potential to alter an individual’s trajectory for future health outcome. The findings from our study suggest healthy nutrition beyond breastfeeding is beneficial for all individuals, with effects becoming non-significant with age. This is demonstrated through the reduction in probability of adult obesity and elevated blood pressure and with improved dietary quality score at one year of age compared to score at three years of age. This observation suggests the critical importance of healthy nutrition in the first 1–2 years of life; however, there may exist limitations with capturing true diet quality given early life dietary scores were computed based on self-reported recall of food intake over a 24-h period.

The World Health Organisation (WHO) advocates exclusive breastfeeding in the first six months of life, and high rates of exclusive breastfeeding have been shown to be associated with benefits to the mother, child and society^15^. The rates of exclusive breastfeeding in the first six months, however, still remain low and far from reaching the global nutrition target for 2025 of at least 50%^16^. We demonstrated that good quality nutrition is beneficial for all individuals and the greatest benefit appears to be most prevalent before one year of age. Additionally, for the first time, we showed that this benefit is differential, i.e. there is greater benefit for breastfeeding in the subset of the population with increased BW-PGS; furthermore the genetic risk score was a better proxy of risk than birthweight.

A recent review on breastfeeding and its potential in reducing the risks of NCDs has reported conflicting data^17^. In addition, meta-analyses of the protective effects of breastfeeding on adult NCDs suggested only modest benefit with limited clinical and public health importance^18,19^. These could indicate that although breastfeeding may have a modest benefit overall, there may be a particular benefit in a subset of the population identified through precision medicine. In a study by Abarin et. al., the duration of exclusive breastfeeding had a differential effect on the trajectory of BMI in childhood, depending on whether they were carriers of the ‘AT’ or ‘AA’ genotype of the FTO variant (rs9939609)^20^. In a separate study, Wu et. al. demonstrated the potential of utilising a polygenic score for BMI to identify and emphasise the importance of exclusive breastfeeding at targeted individuals to reduce the incidence of obesity and its associated non-communicable diseases in both children and adolescents^21^. The polygenic score, developed from a set of variants, mimics fine-mapping by capturing variants which could explain precise genetic and biological mechanisms underlying the phenotype of interest^22^; this provides more power with less bias than single SNP approaches.

Precision medicine, a shift from a one-size-fits-all approach, utilises an individual’s characteristics to guide a clinician in providing the most appropriate treatment at the right time. Precision medicine has been successfully adopted in the field of oncology and reproductive health; however, there has been limited application in life-course health to identify individuals at the highest risk for non-communicable diseases. The life-course approach to health acknowledges crucial and sensitive periods in life, and their relevance in future health outcomes. The first 1000 days in life is one of the early life critical windows for developmental plasticity. Timely introduction of appropriate interventions during sensitive periods will allow maximum benefit on specific health outcomes. The life-course approach hence capitalises on the phenomenon of developmental plasticity where simple and early interventions can be targeted at individuals at increased risk to alter health trajectory for optimal health. We believe that while public health campaigns to the general population should continue, precision medicine provides a way to identify those at highest risk and allows supplemental and targeted intervention to those likely to benefit the most.

The strengths of this study include the availability of a unique prospective longitudinal study cohort, with genetic data and 22 years of phenotype data including nutrition, cardiovascular risk factors and body composition. The Raine Study is representative of the general Western Australian population allowing generalisability to the broader population^23^. Our study, however, was limited by the sample size for detecting small genetic association. The young age and limited variance within the cardiovascular risk factors potentially resulted in the small effect sizes that were seen in our study. Lastly, our findings may not apply to other ethnic populations as most of the study participants were of Caucasian descent.

## Conclusion

Using a longitudinal study population with rich genetic and phenotype data, we have demonstrated the importance of nutrition in the first year of life and, for the first time, the particular benefit of breastfeeding in the highest risk group (as determined by a polygenic risk score for BW) to reduce the risk of later life non-communicable diseases. Our findings indicate that longer duration of breastfeeding is particularly beneficial for individuals with high BW-PGS, leading to lower BMI and SBP in young adults. These data suggest that optimal breastfeeding in the first year of life could offer the greatest benefit to reduce adult disease in those at high genetic risk. This suggests potential for precision medicine in the first 1000 days of life to place individuals on trajectories to better health in adulthood.

## Methods

### Study population

The Raine Study is a prospective pregnancy cohort of 2900 mothers recruited between 1989–1991 (https://www.rainestudy.org.au/)^24^. Recruitment took place at Western Australia’s major perinatal centre, King Edward Memorial Hospital, and nearby private practices. Women who had sufficient English language skills, an expectation to deliver at King Edward Memorial Hospital, and an intention to reside in Western Australia to allow for future follow-up of their child were eligible for the study.

The primary carers (Gen1) completed questionnaires regarding their respective study child, and the children (Gen2) had physical examinations at ages 1, 2, 3, 5, 8, 10, 14, 17, 18, 20, and 22. Ethics approval for the original pregnancy cohort and subsequent follow-ups were granted by the Human Research Ethics Committee of King Edward Memorial Hospital, Princess Margaret Hospital, the University of Western Australia, and the Health Department of Western Australia. Parents, guardians and young adult participants provided written informed consent either before enrolment of at data collection at each follow-up. This study included a subset of the original cohort that had genetic data, were Caucasian, singleton, born at term, and without evidence of fetal anomaly (n = 1328). It was previously demonstrated that the study population is representative of the general population in Western Australia^23^. All research was performed in accordance with the approved guidelines.

### Measures of early life determinants

Gestational age (GA) was determined by either date of last menstrual period (LMP) or fetal biometry at the 18-week gestation ultrasound (USS) examination. If the difference between methods was greater than seven days, GA was derived from USS; otherwise, the LMP method was used. Birthweight was retrieved from hospital records, and percent optimal birthweight (POBW) was calculated as the ratio of observed growth to optimal growth (based on GA, fetal sex, and maternal characteristics including age, parity and height)^25^.

### Early life nutrition

Duration of exclusive breastfeeding and of any breastfeeding (in months) were computed based on self-reported duration of breastfeeding, and the age of the first introduction of other forms of milk and/or solids. The quality of early life nutrition in the first 1000 days of life beyond breastfeeding was assessed at cohort reviews performed at ages one, two and three, through a self-reported detailed recollection of several food groups and beverage intake in the study child over a 24-h period, that was completed by the primary carer and quantified as a dietary score (Eating Assessment in Toddlers (EAT) score—EAT_1_, EAT_2_ and EAT_3_ scores for ages one, two and three, respectively). In brief, the EAT scores were based on the Youth Healthy Eating Index^26^, ranged between 0 to 70, with higher scores indicating healthier dietary quality in the study child. Details of the computation of the EAT scores are described elsewhere^27^. The quality of early life nutrition beyond breastfeeding was assessed through a self-reported detailed recollection of food and beverage intake in the study child over a 24-h period, analysed using the dietary analysis program FoodWorks® (Professional Version 5, 2007, Xyris Software, Brisbane), evaluated and scored by a team of nutritionists. Only EAT scores with a 24-h period recall for dietary intake were valid and used in this study.

### Assessment of adult outcomes

Four areas of adult metabolic syndrome including obesity, hypertension, diabetes, and dyslipidaemia were investigated using appropriate cardiovascular risk factors as surrogates. Obesity was assessed via body mass index (BMI) and DEXA scan measured body composition; hypertension via systolic and diastolic blood pressures (SBP, DBP); diabetes via glucose, insulin and calculated insulin-resistance measured using the homeostasis-model-assessment-for-insulin-resistance (HOMA-IR); and dyslipidaemia via low-density-lipoprotein-cholesterol (LDL-C), total-cholesterol, triglycerides, and high-density-lipoprotein-cholesterol (HDL-C).

All adult outcomes were measured by trained research staff. Height and weight were measured with participants dressed in light clothing. Height was measured (to the nearest 0.1 cm) with the participant standing in the anatomical position, palms facing forward, with shoes off, heels, buttocks and head against the board using a wall-mounted stadiometer, while weight was measured (to the nearest 100 g) using a chair scale. Body mass index (BMI) was calculated using the formula $BMI = \frac{{weight\;({\text{kg}})}}{{height^{2} \;({\text{m}}^{2} )}}$. Body composition was assessed using a Norland XR-36 densitometer (Norland Medical Systems, Inc., Fort Atkinson, WI, USA) which provided estimates of fat mass (in g), lean mass (in g), bone mass (g) and bone area (cm^2^). Total body fat percentage was calculated as $DX_{TFAT} = \frac{{DX_{FATMASS} \left( g \right)}}{{DX_{TOTMASS } \left( g \right)}}*100$. Resting SBP and DBP (mmHg) were measured using an oscillometric sphygmomanometer with an appropriate cuff size for arm circumference. Six sets of readings were recorded, once every 2 min following a 5-min rest period. Average SBP and DBP were calculated after discarding the first set of reading. Blood samples were collected to investigate biochemistry measures including plasma glucose, serum insulin, triglycerides, total-cholesterol, HDL-C and LDL-C. Plasma glucose (mmol/L) was measured using an automated Technicon Axon analyser (Bayer Diagnostics, Sydney, Australia) using a hexokinase method; while serum insulin (mU/ml) was measured using an automated radioimmunoassay (Tosoh, Tokyo, Japan). Homeostasis-model-assessment for Insulin Resistance (HOMA-IR) was calculated by $HOMA - IR = \frac{{Insulin\left( {in mIU/ml} \right)*glucose\left( {in mmol/l} \right)}}{22.5}$. Total-Cholesterol (mmol/L) and triglycerides (mmol/L) were determined enzymatically on the Cobas MIRA analyser (Roche Diagnostics) with reagents from Trace Scientific (Melbourne, Australia), while HDL-C (mmol/L) was determined on a heparin-manganese supernatant. LDL-C (mmol/L) was calculated using the Friedewald formula, valid for Triglycerides < 3.5 mmol/L^28^.

The anthropometric, blood pressure and biochemical outcomes were measured at the 22-year follow-up; body composition was measured at the 20-year follow-up. Biochemical measures were included in this study if participants completed an overnight fast.

### Genetics data

The Raine Study Gen2 participants were genotyped on an Illumina 660 W Quad Array at the Centre for Applied Genomics, Toronto, Canada. Quality control (QC) of the Genome-Wide-Association-Study (GWAS) genotyped data were performed as per standard protocol. In brief, a total of 1593 Raine Study Gen2 participants were genotyped on an Illumina 660 Quad Array, which included 657,366 genetic variants, consisting of ~ 560,000 single-nucleotide-polymorphisms (SNPs) and ~ 95,000 copy number variants (CNVs), at the Centre for Applied Genomics, Toronto, Canada. Plate controls and replicates with a higher proportion of missing data were excluded before individuals were assessed for low genotyping success (> 3% missing), excessive heterozygosity, gender discrepancies between the core data and genotyped data, and cryptic relatedness (π > 0.1875, in between second- and third-degree relatives—e.g. between half-siblings and cousins). At the SNP level, the SNP data were cleaned using plink^29^ following the Wellcome Trust Case—Control Consortium protocol^30^. The exclusion criteria for SNPs included: Hardy–Weinberg-Equilibrium p < 5.7 × 10^–7^; call-rate < 95%; minor-allele-frequency < 1%; and SNPs of possible strand ambiguity (i.e. A/T and C/G SNPs). The cleaned GWAS data were imputed using MACH software^31^ across the 22-autosomes and X-chromosome against the 1000 Genome Project Phase I version 3^32^. A total of 1494 individuals with 535,632 SNPs remained after genotype QC; imputation resulted in 30,061,896 and 1,264,4493 SNPs across the 22-autosomes and X-chromosome, respectively. Principal components (PCs) analysis was carried out, using SMARTPCA from v.3.0 of EIGENSOFT^33^, on the cleaned genotyped data, where PCs were generated for purposes of adjusting for population stratification in all genetic analyses.

### Development of the birthweight polygenic score (BW-PGS)

Genome-wide association analyses have enabled us to identify genetic variants associated with a wide range of traits; however, these effect sizes are usually small with low predictive power^22,34^. Several studies demonstrated increased predictive power with the use of polygenic score (PGS), a metric computed by summing the risk alleles corresponding to the phenotype of interest in each individual, when compared to a small number of genome-wide significant SNPs^11,35^. In addition to identifying and understanding potential aetiology of disease, PGS have also been used to test for genome-wide Gene*Gene and Gene-Environment interactions^11,12^.

As part of a meta-analysis within a large international consortium (n ~ 154,000), we identified 60 SNPs associated with birthweight and adult disease^7^ (Supplementary Table 1). These SNPs data were extracted from the Raine Study Gen2 participants, re-coded to correspond with increasing birthweight, weighted using the beta-coefficients reported in the meta-analysis, summed and re-scaled before the BW-PGS was calculated for each study participant.

### Statistical methods

All measures of adult outcomes and variables in this study were analysed as continuous measures unless otherwise stated. Prior to any analyses, the natural logarithmic-transformation was applied to all adult outcomes which deviated from a normal distribution to approximate normality; duration of breastfeeding was centred at its mean; POBW and BW-PGS were standardised.

Four multivariate linear regression models were fitted to examine the effect of early life nutrition on adult outcomes. In the first model, the effect of early life nutrition was examined after adjusting for POBW, and sex. In the third model, the effect of early life nutrition was examined after adjusting for BW-PGS, sex, and the first two PCs. In models two and four, interaction terms were added to models one and three to investigate effect modification of breastfeeding by POBW and BW-PGS, respectively.

In outcomes where the effect of early life nutrition was moderated by POBW or BW-PGS, breastfeeding, the moderator term (POBW or BW-PGS), and the specific adult outcome were examined as dichotomised variables and analysed using multivariate logistic regression.

Lastly, the potential for the moderation effects of sex on adult outcomes were ascertained by examining the interaction between sex and early life nutrition, and sensitivity analyses were performed by repeating the analyses after imputing the phenotype dataset using Multiple Imputation by Chain Equations (MICE)^36^.

All data were analysed using R and its associated libraries^37^.

## Supplementary Information

Supplementary Information.
