# Supplementary material for: The interactions between genetics and early childhood nutrition influence adult cardiometabolic risk factors
Source: Sci Rep. 2021 Jul 21;11:14826. doi: 10.1038/s41598-021-94206-4 (PMC8295375; doi:10.1038/s41598-021-94206-4)
Supplement: Supplementary file 1 — Supplementary Information. [file 41598_2021_94206_MOESM1_ESM.docx]

# Title Page

**Supplementary Material for**

**TITLE**: The interactions between genetics and early childhood nutrition influence adult cardiometabolic risk factors

Carol A Wang^1,2^

John R Attia^1,2^

Stephen J Lye^3^

Wendy H Oddy^4^

Lawrence Beilin^5^

Trevor A Mori^5^

Claire Meyerkort^6^

Craig E Pennell^1,2^*

^1^ School of Medicine and Public Health, University of Newcastle, New South Wales, Australia

^2^ Hunter Medical Research Institute, New South Wales, Australia

^3^Alliance for Human Development, Lunenfeld-Tanenbaum Research Institute, Sinai Health System, Ontario, Canada

^4^Menzies Institute for Medical Research, University of Tasmania, Hobart, Australia

^5^ Medical School, Royal Perth Hospital Unit, University of Western Australia, Western Australia, Australia

^6^ Sir Charles Gairdner Hospital, Western Australia, Australia

*Corresponding Author

craig.pennell@newcastle.edu.au

**Supplementary Tables**

| **Supplementary Table 1** | SNP list for variants used to develop the BW-PGS |
| --- | --- |
| **Supplementary Table 2** | Association Analyses for body mass index (kg/m^2^) |
| **Supplementary Table 3** | Association Analyses for Obesity  (examining the interaction effects of BW-PGS and the duration of breastfeeding) |
| **Supplementary Table 4** | Association Analyses for Obesity  (examining the effects of dietary quality at one and three years of age) |
| **Supplementary Table 5** | Association analyses for DEXA scan measured percent body fat (at 20 years of age) |
| **Supplementary Table 6** | Sensitivity analyses for body mass index (kg/m^2^)  (Results from pooled imputation analysis) |
| **Supplementary Table 7** | Association Analyses for systolic blood pressure (mmHg)  (examining the effects of dietary quality at one and three years of age) |
| **Supplementary Table 8** | Sensitivity analyses for systolic blood pressure (mmHg)  (Results from pooled imputation analysis) |
| **Supplementary Table 9** | Association Analyses for elevated systolic blood pressure (SBP ≥ 120 mmHg)  (examining the interaction effects of BW-PGS and the duration of breastfeeding) |
| **Supplementary Table 10** | Association Analyses for elevated systolic blood pressure (SBP≥ 120 mmHg)  (examining the effects of dietary quality at one and three years of age) |
| **Supplementary Table 11** | Association analyses for diastolic blood pressure (mmHg) |
| **Supplementary Table 12** | Association Analyses for Fasting Serum Insulin  (examining the effects of dietary quality at one and three years of age) |
| **Supplementary Table 13** | Association analyses for Plasma Glucose_F_ |
| **Supplementary Table 14** | Association analyses for Homeostatic Model Assessment for Insulin Resistance_F_ (HOMA-IR_F_) |
| **Supplementary Table 15** | Association Analyses for Fasting Low-Density-Lipoprotein-Cholesterol (mmol/L)  (examining the effects of dietary quality at one and three years of age) |
| **Supplementary Table 16** | Association analyses for Total Cholesterol_F_ (mmol/L) |
| **Supplementary Table 17** | Association analyses for Triglycerides_F_ (mmol/L) |
| **Supplementary Table 18** | Association analyses for High Density Lipoprotein Cholesterol_F_ (HDL-C_F_) (mmol/L) |
| **Supplementary Table 19** | Three-way interaction association analysis to examine the effect of breastfeeding by POBW and sex for body mass index (kg/m^2^) |
| **Supplementary Table 20** | Three-way interaction association analysis to examine the effect modification of duration of breastfeeding by POBW and sex on systolic blood pressure (mmHg) |
| **Supplementary Table 21** | Three-way interaction association analysis to examine the effect modification of duration of breastfeeding by BW-PGS and sex for body mass index (kg/m^2^) |
| **Supplementary Table 22** | Three-way interaction association analysis to examine the effect modification of duration of breastfeeding by BW-PGS and sex on systolic blood pressure (mmHg) |
| **Supplementary Table 23** | Association Analyses for Body Mass Index (BMI, kg/m^2^)  (examining the interaction effects of sex and the duration of breastfeeding) |
| **Supplementary Table 24** | Association Analyses for Systolic Blood Pressure (SBP, mmHg)  (examining the interaction effects of sex and the duration of breastfeeding) |

Supplementary Table 1. SNP list for variants used to develop the BW-PGS

| Locus | SNP | CHR | BP | EFFECT  ALLELE | OTHER  ALLELE | EAF | BETA | SE | P_value |
| --- | --- | --- | --- | --- | --- | --- | --- | --- | --- |
| WNT4-ZBTB40 | rs2473248 | 1 | 22536643 | C | T | 0·889 | 0·033 | 0·006 | 1·1x10-8 |
| ZBTB7B | rs3753639 | 1 | 154986091 | C | T | 0·2285 | 0·031 | 0·004 | 7·3x10-12 |
| DTL | rs61830764 | 1 | 212289976 | A | G | 0·3513 | 0·022 | 0·004 | 5·6x10-8 |
| FCGR2B | rs72480273 | 1 | 161644871 | C | A | 0·1848 | 0·031 | 0·005 | 8·0x10-10 |
| EPAS1 | rs1374204 | 2 | 46484205 | T | C | 0·6829 | 0·047 | 0·004 | 6·2x10-29 |
| ATAD2B | rs7575873 | 2 | 23962647 | A | G | 0·8737 | 0·038 | 0·006 | 1·3x10-11 |
| CPA3 | rs10935733 | 3 | 148622968 | T | C | 0·4422 | 0·022 | 0·004 | 9·2x10-9 |
| ADCY5 | rs11719201 | 3 | 123068744 | T | C | 0·2281 | 0·046 | 0·004 | 2·4x10-26 |
| CCNL1-LEKR1 | rs13322435 | 3 | 156795468 | A | G | 0·431 | 0·053 | 0·004 | 3·7x10-41 |
| PTH1R | rs2242116 | 3 | 46941116 | A | G | 0·3756 | 0·022 | 0·004 | 1·4x10-8 |
| HHIP | rs6537307 | 4 | 145601863 | G | A | 0·4957 | 0·025 | 0·004 | 9·5x10-12 |
| LCORL | rs925098 | 4 | 17919811 | G | A | 0·2701 | 0·034 | 0·004 | 5·4x10-16 |
| EBF1 | rs7729301 | 5 | 157886953 | A | G | 0·7156 | 0·024 | 0·004 | 1·6x10-8 |
| 5q11.2 | rs854037 | 5 | 57091783 | A | G | 0·8214 | 0·027 | 0·005 | 2·2x10-8 |
| ESR1 | rs1101081 | 6 | 152032917 | C | T | 0·7629 | 0·038 | 0·004 | 1·6x10-19 |
| L3MBTL3 | rs1415701 | 6 | 130345835 | G | A | 0·7541 | 0·025 | 0·004 | 2·6x10-9 |
| CDKAL1 | rs35261542 | 6 | 20675792 | C | A | 0·7448 | 0·044 | 0·004 | 4·4x10-27 |
| HMGA1 | rs7742369 | 6 | 34165721 | G | A | 0·1817 | 0·028 | 0·005 | 1·0x10-8 |
| HIST1H2BE | rs9379832 | 6 | 26186200 | A | G | 0·6946 | 0·023 | 0·004 | 6·6x10-8 |
| IGF2BP3 | rs11765649 | 7 | 23479013 | T | C | 0·753 | 0·027 | 0·004 | 5·8x10-10 |
| YKT6-GCK | rs138715366 | 7 | 44246271 | C | T | 0·9885 | 0·241 | 0·023 | 7·2x10-26 |
| MLXIPL | rs62466330 | 7 | 73056805 | C | T | 0·0604 | 0·049 | 0·008 | 1·2x10-12 |
| TBX20 | rs6959887 | 7 | 35295365 | A | G | 0·6185 | 0·023 | 0·004 | 1·5x10-9 |
| GNA12 | rs798489 | 7 | 2801803 | C | T | 0·755 | 0·023 | 0·004 | 2·0x10-8 |
| SLC45A4 | rs12543725 | 8 | 142247979 | G | A | 0·5898 | 0·023 | 0·004 | 1·2x10-9 |
| ANK1-NKX6-3 | rs13266210 | 8 | 41533514 | A | G | 0·7962 | 0·031 | 0·005 | 1·3x10-11 |
| TRIB1 | rs6989280 | 8 | 126508746 | G | A | 0·7179 | 0·022 | 0·004 | 2·2x10-7 |
| LPAR1 | rs2150052 | 9 | 113945067 | T | A | 0·49636 | 0·021 | 0·004 | 2·2x10-8 |
| PTCH1 | rs28510415 | 9 | 98245026 | G | A | 0·08407 | 0·056 | 0·007 | 1·5x10-17 |
| STRBP | rs700059 | 9 | 125824055 | G | A | 0·15355 | 0·033 | 0·005 | 4·7x10-10 |
| PHF19 | rs7847628 | 9 | 123631225 | G | A | 0·68935 | 0·023 | 0·004 | 1·0x10-8 |
| PLEKHA1 | rs2421016 | 10 | 124167512 | T | C | 0·47441 | 0·021 | 0·004 | 1·8x10-8 |
| HHEX-IDE | rs61862780 | 10 | 94468643 | T | C | 0·54487 | 0·028 | 0·004 | 3·0x10-14 |
| ADRB1 | rs7076938 | 10 | 115789375 | T | C | 0·74435 | 0·036 | 0·004 | 4·7x10-18 |
| NT5C2 | rs74233809 | 10 | 104913940 | C | T | 0·07945 | 0·037 | 0·007 | 5·2x10-8 |
| MTNR1B | rs10830963 | 11 | 92708710 | G | C | 0·25784 | 0·023 | 0·004 | 2·9x10-8 |
| INS-IGF2 | rs72851023 | 11 | 2130620 | T | C | 0·06912 | 0·048 | 0·008 | 2·9x10-10 |
| APOLD1 | rs11055034 | 12 | 12890626 | C | A | 0·69934 | 0·022 | 0·004 | 1·8x10-7 |
| ITPR2 | rs12823128 | 12 | 26872730 | T | C | 0·53331 | 0·021 | 0·004 | 1·9x10-8 |
| HMGA2 | rs1351394 | 12 | 66351826 | T | C | 0·47709 | 0·044 | 0·004 | 1·9x10-32 |
| ABCC9 | rs139975827 | 12 | 22068161 | G | A | 0·58773 | 0·025 | 0·004 | 1·1x10-8 |
| IGF1 | rs7964361 | 12 | 102994878 | A | G | 0·09501 | 0·039 | 0·007 | 4·7x10-9 |
| RNF219-AS1 | rs1819436 | 13 | 78580283 | C | T | 0·8636 | 0·033 | 0·006 | 6·3x10-9 |
| LINC00332 | rs2324499 | 13 | 40662001 | G | C | 0·67402 | 0·022 | 0·004 | 7·3x10-8 |
| RB1 | rs2854355 | 13 | 48882363 | G | A | 0·29964 | 0·023 | 0·004 | 9·8x10-8 |
| FES | rs12906125 | 15 | 91427612 | G | A | 0·69747 | 0·023 | 0·004 | 1·7x10-8 |
| IGF1R | rs7402982 | 15 | 99193269 | A | G | 0·43915 | 0·023 | 0·004 | 2·3x10-9 |
| GPR139 | rs1011939 | 16 | 19992996 | G | A | 0·30875 | 0·022 | 0·004 | 1·3x10-7 |
| CLDN7 | rs113086489 | 17 | 7171356 | T | C | 0·44908 | 0·031 | 0·004 | 9·1 x10-16 |
| SP6-SP2 | rs12942207 | 17 | 45968294 | C | T | 0·29883 | 0·022 | 0·004 | 5·1x10-8 |
| SUZ12P1-CRLF3 | rs144843919^ | 17 | 29037339 | G | A | 0·97222 | 0·066 | 0·012 | 1·4x10-8 |
| PEPD | rs10402712 | 19 | 33926013 | A | G | 0·27166 | 0·022 | 0·004 | 4·4x10-7 |
| ACTL9 | rs61154119 | 19 | 8787750 | T | G | 0·83999 | 0·028 | 0·005 | 1·1x10-7 |
| C20orf203 | rs28530618 | 20 | 31275581 | A | G | 0·48052 | 0·026 | 0·004 | 7·7x10-12 |
| MAFB | rs6016377 | 20 | 39172728 | T | C | 0·43974 | 0·024 | 0·004 | 9·5x10-10 |
| JAG1 | rs6040076 | 20 | 10658882 | C | G | 0·49462 | 0·023 | 0·004 | 2·0x10-9 |
| NRIP1 | rs2229742 | 21 | 16339172 | G | C | 0·89414 | 0·036 | 0·006 | 2·2x10-9 |
| KREMEN1 | rs134594 | 22 | 29468456 | C | T | 0·34844 | 0·023 | 0·004 | 1·0x10-8 |
| SREBF2 | rs62240962 | 22 | 42259524 | C | T | 0·92035 | 0·047 | 0·007 | 9·7x10-12 |
| PLAC1 | rs11096402^ | X | 133827868 | G | A | 0·5528 | 0·028 | 0·005 | 1·3x10-9 |

^denotes variant not used in the development of the BW-PGS due to poor imputation score; CHR – Chromosome number; BP – Base position, EFFECT ALLELE – Allele corresponding to increasing birth weight; OTHER ALLELE- Allele corresponding to decreasing birth weight; EAF – Effect allele frequency; BETA- Beta coefficient from GWAS meta-analysis of birth weight; SE – Standard error from GWAS meta-analysis of birth weight; P-value - P-value from GWAS meta-analysis of birth weight

Supplementary Table 2. Association Analyses for body mass index (kg/m^2^)

|  | Model 1 ^ε^ | |
| --- | --- | --- |
| Predictors | Estimate  (95% CI) | P |
| Intercept | 25·19  (24·61 – 25·78) | **<0·001** |
| BW-PGS ^§^ | 0·18  (-0·25 – 0·61) | 0·41 |
| Duration BF  (months) ^†^ | -0·05  (-0·11 – 0·01) | 0.11 |
| Sex  (M) | 0·24  (-0·58 – 1·06) | 0·57 |
| EAT_1_ score ^‡^ | -0·57  (-0·99 – -0·14) | **0·0090** |
| BW-PGS * Duration BF | -0·06  (-0·11 – -0·00) | **0·037** |
|  | Model 2 ^φ^ | |
| Predictors | Estimate  (95% CI) | P |
| Intercept | 25·14  (24·52 – 25.75) | **<0·001** |
| BW-PGS ^§^ | 0·36  (-0·09 – 0·81) | 0·11 |
| Duration BF  (months) ^†^ | -0·29  (-0·09 –0·03) | 0·34 |
| Sex  (M) | 0·11  (-0·74 – 0·97) | 0·80 |
| EAT_3_ score ^ǂ^ | -0·46  (-0·91 – -0·02) | **0·043** |
| BW-PGS * Duration BF | -0·08  (-0·13 – -0·03) | **0·0040** |

^§^ BW-PGS = birth weight polygenic score (standardised); ^†^ Duration of BF = Duration of any breastfeeding (mean-centred); ^‡^ EAT_1_ score (standardised) = quality of early life nutrition in first year of life; ^δ^ adjustment for population stratification; ^ǂ^ EAT_3_ score (standardised) = quality of early life nutrition in third year of life; ^ε^ Model 1 examines the effect of duration of breastfeeding and EAT_1_ score; ^φ^ Model 2 examine the effect of duration of breastfeeding and EAT_3_ score; All models adjusting for BW-PGS, sex and the first two principal components [PCs] (estimates for PCs not presented)

Supplementary Table 3. Association Analyses for Obesity

| Overweight  (BMI ≥ 25 kg/m^2^) | Model 1 ^ε^ | | Model 2 ^φ^ | |
| --- | --- | --- | --- | --- |
| Predictors | Log Odds  (95% CI) | P | Log Odds  (95% CI) | P |
| Intercept | 0·28  (-0·50 – 1·07) | 0·48 | 0·12  (-0·68 – 0·91) | 0·77 |
| BW-PGS (High Score) ^§^ | 0·13  (-0·29 – 0·54) | 0·54 | 1·01  (0·35 – 1·71) | **0·0033** |
| Duration BF (≥ 6 months) | -0·39  (-0·75 – -0·04) | **0·031** | -0·07  (-0·48 – 0·34) | 0·72 |
| Sex  (M) | 0·42  (0·08 – 0·76) | **0·016** | 0·48  (0·14 – 0·83) | **0·0063** |
| EAT_1_ score ^‡^ | -0·02  (-0·04 – 0·00) | **0·055** | -0·02  (-0·04 – -0·00) | **0·039** |
| BW-PGS (High Score) *  Duration BF (≥ 6 months) |  |  | -1·50  (-2·40 – -0·62) | **0·00096** |
| Obese  (BMI ≥ 30 kg/m^2^) | Model 3 ^ε^ | | Model 4 ^φ^ | |
| Predictors | Log Odds  (95% CI) | P | Log Odds  (95% CI) | P |
| Intercept | -0·08  (-1·17 – 1.00) | 0·88 | -0·25  (-1·35 – 0·84) | 0·65 |
| BW-PGS (High Score) ^§^ | -0·19  (-0·83 – 0·40) | 0·54 | 0·60  (-0·19 – 1·35) | 0·13 |
| Duration BF (≥ 6 months) | -0·59  (-1·08 – -0·10) | **0·018** | -0·24  (-0·79 – 0·31) | 0·39 |
| Sex  (M) | -0·40  (-0·89 – 0·08) | 0·10 | -0·340  (-0·83 – 0·15) | 0·17 |
| EAT_1_ score ^‡^ | -0·03  (-0·05 – -0·00) | **0·030** | -0·03  (-0·06 – -0·00) | **0·024** |
| BW-PGS (High Score) *  Duration BF (≥ 6 months) |  |  | -2·23  (-4·18 – -0·75) | **0·0079** |

^§^ BW-PGS (High Score) = birthweight polygenic score ≥ 80^th^ percentile of BW-PGS; ^‡^ EAT_1_ score = quality of early life nutrition in first year of life; ^δ^ adjustment for population stratification; ^ε^ Models 1 and 3 examine the effect of duration of breastfeeding on the risk of being overweight and obese, respectively, adjusting for BW-PGS, sex and EAT_1_ score; ^φ^ Models 2 and 4 examines the effect modification of duration of breastfeeding by BW-PGS on the risk of being overweight and obese, respectively, adjusting for sex and EAT_1_ score; All models adjusting for BW-PGS, sex and the first two principal components [PCs] (estimates for PCs not presented)

Supplementary Table 4. Association Analyses for Obesity

|  | Model 1 ^α^  (examining overweight, BMI ≥ 25 kg/m^2^, as an outcome) | | Model 2 ^β^  (examining obese, BMI ≥ 25 kg/m^2^, as an outcome) | |
| --- | --- | --- | --- | --- |
| Predictors | Log Odds  (95% CI) | P | Log Odds  (95% CI) | P |
| Intercept | -0·69  (-1·06 – -0·32) | 0·48 | -1·50  (-1·97 – -1·02) | **<0·001** |
| BW-PGS (High Score) ^§^ | 1·01  (0·34 – 1·69) | 0·54 | 0·60  (-0·17 – 1·36) | 0·13 |
| Duration BF (≥ 6 months) | -0·07  (-0·48 – 0·33) | **0·031** | -0·24  (-0·79 – 0·31) | 0·39 |
| Sex  (M) | 0·48  (0·14 – 0·83) | **0·016** | -0·34  (-0·83 – 0·15) | 0·17 |
| EAT_1_ score ^‡^  (standardised) | -0·19  (-0·37 – -0·01) | **0·055** | -0·29  (-0·54 – -0·04) | **0·024** |
| BW-PGS (High Score) *  Duration BF (≥ 6 months) | -1·50  (-2·38 – -0·61) | **0·001** | -2·23  (-3·87 – -0·58) | **0·0080** |
|  | Model 3 ^ε^  (examining overweight, BMI ≥ 25 kg/m^2^ , as an outcome) | | Model 4 ^φ^  (examining obese, BMI ≥ 30 kg/m^2^ , as an outcome) | |
| Predictors | Log Odds  (95% CI) | P | Log Odds  (95% CI) | P |
| Intercept | -0·87  (-1·27 – -0.47) | **<0·001** | -1·59  (-2·10 – -1·07) | **<0·001** |
| BW-PGS (High Score) ^§^ | 1·19  (0·48 – 1·91) | 0·001 | 0·61  (-0·22 – 1·44) | 0·15 |
| Duration BF (≥ 6 months) | 0·13  (-0·30 – 0·55) | 0·56 | -0·13  (-0·71 – 0·45) | 0·65 |
| Sex  (M) | 0·45  (0·09 – 0·81) | **0·014** | -0·32  (-0·83 – 0·20) | 0·23 |
| EAT_3_ score ^ǂ^  (standardised) | -0·04  (-0·22 – 0·15) | 0·70 | -0·25  (-0·52 – 0·02) | 0·066 |
| BW-PGS (High Score) *  Duration BF (≥ 6 months) | -1·62  (-2·55 – -0·69) | **0·0010** | -2·84  (-5·01 – -0·67) | **0·010** |

^§^ BW-PGS (High score)= birthweight polygenic score ≥ 80^th^ percentile of BW-PGS; ^‡^ EAT_1_ score (standardised) = quality of early life nutrition in first year of life; ^δ^ adjustment for population stratification; ^ǂ^ EAT_3_ score (standardised) = quality of early life nutrition in third year of life; ^α^ Model 1 examines the effect of duration of breastfeeding and EAT_1_ score on the risk of being overweight; ^β^ Model 2 examines the effect of duration of breastfeeding and EAT_1_ score on the risk of being obese; ^ε^ Model 3 examines the effect of duration of breastfeeding and EAT_3_ score on the risk of being overweight; ^φ^ Model 4 examines the effect of duration of breastfeeding and EAT_3_ score on the risk of being obese; All models adjusting for BW-PGS, sex and the first two principal components [PCs] (estimates for PCs not presented)

Supplementary Table 5. Association analyses for percent body fat (at 20 years of age) by DEXA

| Percent Optimal Birth Weight (POBW) | Model 1 ^α^ | | Model 2 ^β^ | |
| --- | --- | --- | --- | --- |
| Predictors | Estimate  (95% CI) | P | Estimate  (95% CI) | P |
| Intercept | 43·69  (40·68 – 46·70) | **<0·0001** | 43·69  (40·67 – 46·71) | **<0·0001** |
| POBW ^γ^ | 0·31  (-0·34 – 0·97) | 0·35 | 0·31  (-0·34 – 0·97) | 0·35 |
| Duration BF  (months) ^†^ | -0·10  (-0·20 – -0·01) | **0·033** | -0·10  (-0·20 – -0·01) | **0·034** |
| Sex  (M) | -17·94  (-19·24 – -16·65) | **<0·0001** | -17·95  (-19·25 – -16·65) | **<0·0001** |
| EAT_1_ score ^‡^ | -0·10  (-0·16 – -0·03) | **0·0047** | -0·10  (-0·16 – -0·03) | **0·0047** |
| POBW * Duration BF |  |  | -0·00  (-0·08 – 0·08) | 0·097 |
| Birthweight Polygenic Score  (BW-PGS) | Model 3 ^ε^ | | Model 4 ^φ^ | |
| Predictors | Estimate  (95% CI) | P | Estimate  (95% CI) | P |
| Intercept | 43·73  (40·71 – 46·75) | **<0·0001** | 43·57  (40·55 – 46·59) | **<0·0001** |
| BW-PGS ^§^ | 0·00  (-0·65 – 0·65) | 1·0 | 0·07  (-0·58 – 0·72) | 0·83 |
| Duration BF  (months) ^†^ | -0·09  (-0·19 – 0·00) | 0·056 | -0·09  (-0·19 – 0·00) | 0·054 |
| Sex  (M) | -17·91  (-19·20 – -16·62) | **<0·0001** | -17·84  (-19·14 – -16·55) | **<0·0001** |
| EAT_1_ score ^‡^ | -0·10  (-0·16 – -0·03) | **0·0042** | -0·09  (-0·16 – -0·03) | **0·0054** |
| BW-PGS * Duration BF |  |  | -0·08  (-0·17 – 0·01) | 0·069 |

^γ^ POBW = Percent Optimal Birth Weight (standardised)^†^ Duration of BF = Duration of any breastfeeding (mean-centred); ^‡^ EAT_1_ score = quality of early life nutrition in first year of life; ^§^ BW-PGS = birth weight polygenic score (standardised); ^δ^ adjustment for population stratification; ^α^ Model 1 examines the effect of duration of breastfeeding adjusting for POBW, sex and EAT_1_ score; ^β^ Model 2 examines the effect modification of duration of breastfeeding by POBW adjusting for sex and EAT_1_ score; ^ε^ Model 3 examines the effect of duration of breastfeeding adjusting for BW-PGS, sex, EAT_1_ score and the first two principal components [PCs] (estimates not presented); ^φ^ Model 4 examines the effect modification of duration of breastfeeding by BW-PGS adjusting for sex, EAT_1_ score and the first two principal components [PCs] (estimates not presented)

Supplementary Table 6. Sensitivity analyses for body mass index (kg/m^2^)

(Results from pooled imputation analysis)

| Percent Optimal Birth Weight (POBW) | Model 1 ^α^ | | Model 2 ^β^ | |
| --- | --- | --- | --- | --- |
| Predictors | Estimate  (SE) | P | Estimate  (SE) | P |
| Intercept | 28·26 (0·79) | **<0·0001** | 28·26 (0·79) | **<0·0001** |
| POBW ^γ^ | 0·35 (0·16) | **0·033** | 0·35 (0·16) | **0·032** |
| Duration BF  (months) ^†^ | -0·05 (0·02) | **0·032** | -0·05 (0·02) | **0·032** |
| Sex  (M) | 0·11 (0·34) | 0·74 | 0·11 (0·34) | 0·74 |
| EAT_1_ score ^‡^ | -0·07 (0·02) | **0·00021** | -0·07 (0·02) | **0·00021** |
| POBW * Duration BF |  |  | 0·00 (0·02) | 0·93 |
| Birthweight Polygenic Score  (BW-PGS) | Model 3 ^ε^ | | Model 4 ^φ^ | |
| Predictors | Estimate  (SE) | P | Estimate  (SE) | P |
| Intercept | 28·36 (0·80) | **<0·0001** | 28·30 (0·79) | **<0·0001** |
| BW-PGS ^§^ | 0·06 (0·18) | 0·74 | 0·07 (0·18) | 0·71 |
| Duration BF  (months) ^†^ | -0·05 (0·02) | 0·058 | -0·05 (0·02) | 0·053 |
| Sex  (M) | 0·11 (0·34) | 0·74 | 0·14 (0·34) | 0·69 |
| EAT_1_ score ^‡^ | -0·07 (0·02) | **0·00015** | -0·07 (0·02) | **0·00017** |
| BW-PGS * Duration BF |  |  | -0·05 (0·02) | **0·038** |

^γ^ POBW = Percent Optimal Birth Weight (standardised)^†^ Duration of BF = Duration of any breastfeeding (mean-centred); ^‡^ EAT_1_ score = quality of early life nutrition in first year of life; ^§^ BW-PGS = birth weight polygenic score (standardised); ^δ^ adjustment for population stratification; ^α^ Model 1 examines the effect of duration of breastfeeding adjusting for POBW, sex and EAT_1_ score; ^β^ Model 2 examines the effect modification of duration of breastfeeding by POBW adjusting for sex and EAT_1_ score; ^ε^ Model 3 examines the effect of duration of breastfeeding adjusting for BW-PGS, sex, EAT_1_ score and the first two principal components [PCs] (estimates not presented); ^φ^ Model 4 examines the effect modification of duration of breastfeeding by BW-PGS adjusting for sex, EAT_1_ score the first two principal components [PCs] (estimates not presented)

Supplementary Table 7. Association Analyses for systolic blood pressure (mmHg)

|  | Model 1 ^ε^ | |
| --- | --- | --- |
| Predictors | Estimate  (95% CI) | P |
| Intercept | 113·77  (112·65 – 114·89) | **<0·001** |
| BW-PGS ^§^ | 0·20  (-0·62 – 1·02) | 0·63 |
| Duration BF  (months) ^†^ | 0·12  (0·01 – 0·23) | **0.041** |
| Sex  (M) | 9·59  (8·01 – 11·16) | **<0·001** |
| EAT_1_ score ^‡^ | -0·97  (-1·78 – -0·16) | **0·020** |
| BW-PGS * Duration BF | -0·12  (-0·22 – -0·01) | **0·030** |
|  | Model 2 ^φ^ | |
| Predictors | Estimate  (95% CI) | P |
| Intercept | 113·71  (112·49 – 114.92) | **<0·001** |
| BW-PGS ^§^ | 0·34  (-0·54 – 1·21) | 0·45 |
| Duration BF  (months) ^†^ | 0·10  (-0·01 – 0·22) | 0·086 |
| Sex  (M) | 9·35  (7·66 – 11·03) | **<0·001** |
| EAT_3_ score ^ǂ^ | 0·29  (-0·58 – 1·17) | 0·51 |
| BW-PGS * Duration BF | -0·15  (-0·25 – -0·04) | **0·0080** |

^§^ BW-PGS = birth weight polygenic score (standardised); ^†^ Duration of BF = Duration of any breastfeeding (mean-centred); ^‡^ EAT_1_ score (standardised) = quality of early life nutrition in first year of life; ^δ^ adjustment for population stratification; ^ǂ^ EAT_3_ score (standardised) = quality of early life nutrition in third year of life; ^ε^ Model 1 examines the effect of duration of breastfeeding and EAT_1_ score; ^φ^ Model 2 examine the effect of duration of breastfeeding and EAT_3_ score; All models adjusting for BW-PGS, sex and the first two principal components [PCs] (estimates not presented)

Supplementary Table 8. Sensitivity analyses for systolic blood pressure (mmHg)

(Results from pooled imputation analysis)

| Percent Optimal Birth Weight (POBW) | Model 1 ^α^ | | Model 2 ^β^ | |
| --- | --- | --- | --- | --- |
| Predictors | Estimate  (SE) | P | Estimate  (SE) | P |
| Intercept | 117·34 (1·76) | **<0·0001** | 117·31 (1·77) | **<0·0001** |
| POBW ^γ^ | -0·41 (0·35) | 0·24 | -0·41 (0·35) | 0·24 |
| Duration BF  (months) ^†^ | 0·10 (0·05) | 0·059 | 0·10 (0·05) | 0·065 |
| Sex  (M) | 9·72 (0·70) | **<0·0001** | 9·74 (0·70) | **<0·0001** |
| EAT_1_ score ^‡^ | -0·09 (0·04) | **0·024** | -0·09 (0·04) | **0·025** |
| POBW * Duration BF |  |  | 0·02 (0·04) | 0·71 |
| Birthweight Polygenic Score  (BW-PGS) | Model 3 ^ε^ | | Model 4 ^φ^ | |
| Predictors | Estimate  (SE) | P | Estimate  (SE) | P |
| Intercept | 117·39 (1·77) | **<0·0001** | 117·31 (1·78) | **<0·0001** |
| BW-PGS ^§^ | 0·05 (0·37) | 0·89 | 0·06 (0·37) | 0·88 |
| Duration BF  (months) ^†^ | 0·10 (0·05) | 0·059 | 0·10 (0·05) | 0·063 |
| Sex  (M) | 9·75 (0·70) | **<0·0001** | 9·79 (0·70) | **<0·0001** |
| EAT_1_ score ^‡^ | -0·09 (0·04) | **0·023** | -0·09 (0·04) | **0·026** |
| BW-PGS * Duration BF |  |  | -0·07 (0·05) | 0·15 |

^γ^ POBW = Percent Optimal Birth Weight (standardised)^†^ Duration of BF = Duration of any breastfeeding (mean-centred); ^‡^ EAT_1_ score = quality of early life nutrition in first year of life; ^§^ BW-PGS = birth weight polygenic score (standardised); ^δ^ adjustment for population stratification; ^α^ Model 1 examines the effect of duration of breastfeeding adjusting for POBW, sex and EAT_1_ score; ^β^ Model 2 examines the effect modification of duration of breastfeeding by POBW adjusting for sex and EAT_1_ score; ^ε^ Model 3 examines the effect of duration of breastfeeding adjusting for BW-PGS, sex, EAT_1_ score and the first two principal components [PCs] (estimates not presented); ^φ^ Model 4 examines the effect modification of duration of breastfeeding by BW-PGS adjusting for sex, EAT_1_ score the first two principal components [PCs] (estimates not presented)

Supplementary Table 9. Association Analyses for elevated systolic blood pressure (SBP ≥ 120mmHg)

| SBP ≥ 120 mmHg | Model 1 ^ε^ | | Model 2 ^φ^ | |
| --- | --- | --- | --- | --- |
| Predictors | Log Odds  (95% CI) | P | Log Odds  (95% CI) | P |
| Intercept | -0·39  (-1·16 – 0·37) | 0·31 | -0·62  (-1·41 – 0·16) | 0·12 |
| BW-PGS (High Score) ^§^ | 0·03  (-0·38 – 0·44) | 0·88 | 0·83  (0·18 – 1·49) | **0·012** |
| Duration BF (≥ 6 months) ^†^ | 0·19  (-0·17 – 0·55) | 0·31 | 0·47  (0·07 – 0·88) | **0·023** |
| Sex  (M) | 1·53  (1·20 – 1·88) | **<0·0001** | 1·61  (1·27 – 1·96) | **<0·0001** |
| EAT_1_ score ^‡^ | -0·02  (-0·04 – -0·00) | **0·021** | -0·02  (-0·04 – -0·00) | **0·022** |
| BW-PGS (High Score) *  Duration BF (≥ 6 months) |  |  | -1·34  (-2·20 – -0·49) | **0·0022** |

^†^ Duration of BF = Duration of any breastfeeding (mean-centred); ^‡^ EAT_1_ score = quality of early life nutrition in first year of life; ^§^ BW-PGS = birth weight polygenic score (standardised); ^δ^ adjustment for population stratification; ^ε^ Model 1 examines the effect of duration of breastfeeding adjusting for BW-PGS, sex and EAT_1_ score; ^φ^ Model 2 examines the effect modification of duration of breastfeeding by BW-PGS adjusting for sex and EAT_1_ score; All models adjusting for BW-PGS, sex and the first two principal components [PCs] (estimates not presented)

Supplementary Table 10. Association Analyses for elevated systolic blood pressure (SBP≥ 120 mmHg)

|  | Model 1 ^α^ | |
| --- | --- | --- |
| Predictors | Log Odds  (95% CI) | P |
| Intercept | -1·50  (-1·89 – -1·11) | **<0·001** |
| BW-PGS (High Score) ^§^ | 0·83  (0·18 – 1·48) | **0·012** |
| Duration BF (≥ 6 months) | 0·47  (0·07 – 0·88) | **0·023** |
| Sex  (M) | 1·61  (1·26 – 1·95) | **<0·001** |
| EAT_1_ score ^‡^ | -0·20  (-0·38 – -0·03) | **0·022** |
| BW-PGS (High Score) *  Duration BF (≥ 6 months) | -1·34  (-2·19 – -0·48) | **0·0022** |
|  | Model 2 ^ε^ | |
| Predictors | Log Odds  (95% CI) | P |
| Intercept | -1·51  (-1·92 – -1.10) | **<0·001** |
| BW-PGS (High Score) ^§^ | 1·13  (0·45 – 1·82) | **0·001** |
| Duration BF (≥ 6 months) | 0·49  (0·06 – 0·91) | **0·024** |
| Sex  (M) | 1·53  (1·17 – 1·89) | **<0·001** |
| EAT_3_ score ^ǂ^ | -0·07  (-0·25 – 0·12) | 0·47 |
| BW-PGS (High Score) *  Duration BF (≥ 6 months) | -1·68  (-2·56 – -0·78) | **<0·001** |

^§^ BW-PGS (High score)= birthweight polygenic score ≥ 80^th^ percentile of BW-PGS; ^‡^ EAT_1_ score (standardised) = quality of early life nutrition in first year of life; ^δ^ adjustment for population stratification; ^ǂ^ EAT_3_ score (standardised) = quality of early life nutrition in third year of life; ^α^ Model 1 examines the effect of duration of breastfeeding and EAT_1_ score on the risk of SBP ≥ 120mmHg; ^ε^ Model 2 examines the effect of duration of breastfeeding and EAT_3_ score on the risk of SBP ≥ 120mmHg; All models adjusting for BW-PGS, sex and the first two principal components [PCs] (estimates not presented)

Supplementary Table 11. Association analyses for diastolic blood pressure (mmHg)

| Percent Optimal Birth Weight (POBW) | Model 1 ^α^ | | Model 2 ^β^ | |
| --- | --- | --- | --- | --- |
| Predictors | Estimate  (95% CI) | P | Estimate  (95% CI) | P |
| Intercept | 69·43  (66·89 – 71·96) | **<0·0001** | 69·31  (66·76 – 71·85) | **<0·0001** |
| POBW ^γ^ | -0·08  (-0·64 – 0·47) | 0·77 | -0·11  (-0·67 – 0·45) | 0·70 |
| Duration BF  (months) ^†^ | 0·08  (0·00 – 0·16) | **0·046** | 0·08  (-0·00 – 0·15) | 0·060 |
| Sex  (M) | -0·26  (-1·35 – 0·82) | 0·63 | -0·18  (-1·28 – 0·91) | 0·74 |
| EAT_1_ score ^‡^ | -0·05  (-0·11 – 0·00) | 0·060 | -0·05  (-0·11 – 0·00) | 0·067 |
| POBW * Duration BF |  |  | 0·04  (-0·03 – 0·10) | 0·30 |
| Birthweight Polygenic Score  (BW-PGS) | Model 3 ^ε^ | | Model 4 ^φ^ | |
| Predictors | Estimate  (95% CI) | P | Estimate  (95% CI) | P |
| Intercept | 69·65  (67·11 – 72·19) | **<0·0001** | 69·56  (67·02 – 72·10) | **<0·0001** |
| BW-PGS ^§^ | -0·24  (-0·80 – 0·32) | 0·40 | -0·19  (-0·76 – 0·37) | 0·51 |
| Duration BF  (months) ^†^ | 0·08  (0·00 – 0·16) | **0·039** | 0·08  (0·01 – 0·16) | **0·036** |
| Sex  (M) | -0·29  (-1·37 – 0·80) | 0·60 | -0·25  (-1·33 – 0·83) | 0·65 |
| EAT_1_ score ^‡^ | -0·06  (-0·11 – -0·00) | **0·043** | -0·06  (-0·11 – -0·00) | **0·049** |
| BW-PGS * Duration BF |  |  | -0·04  (-0·11 – 0·03) | 0·25 |

^γ^ POBW = Percent Optimal Birth Weight (standardised)^†^ Duration of BF = Duration of any breastfeeding (mean-centred); ^‡^ EAT_1_ score = quality of early life nutrition in first year of life; ^§^ BW-PGS = birth weight polygenic score (standardised); ^δ^ adjustment for population stratification; ^α^ Model 1 examines the effect of duration of breastfeeding adjusting for POBW, sex and EAT_1_ score; ^β^ Model 2 examines the effect modification of duration of breastfeeding by POBW adjusting for sex and EAT_1_ score; ^ε^ Model 3 examines the effect of duration of breastfeeding adjusting for BW-PGS, sex, EAT_1_ score and the first two principal components [PCs] (estimates not presented); ^φ^ Model 4 examines the effect modification of duration of breastfeeding by BW-PGS adjusting for sex, EAT_1_ score and the first two principal components [PCs] (estimates not presented)

Supplementary Table 12. Association Analyses for Fasting Serum Insulin (Natural Log Transformed)

|  | Model 1 ^ε^ | |
| --- | --- | --- |
| Predictors | Estimate  (95% CI) | P |
| Intercept | 2·10  (2·05 – 2·15) | **<0·001** |
| BW-PGS ^§^ | -0·02  (-0·05 – 0·02) | 0·43 |
| Duration BF  (months) ^†^ | -0·00  (-0·01 – 0·00) | 0.13 |
| Sex  (M) | -0·19  (-0·26 – -0·11) | **<0·001** |
| EAT_1_ score ^‡^ | -0·04  (-0·08 – 0·00) | 0·066 |
| BW-PGS * Duration BF | 0·00  (-0·00 – 0·01) | 0·81 |
|  | Model 2 ^φ^ | |
| Predictors | Estimate  (95% CI) | P |
| Intercept | 2·10  (2·04 – 2·15) | **<0·001** |
| BW-PGS ^§^ | -0·02  (-0·06 – 0·03) | 0·46 |
| Duration BF  (months) ^†^ | -0·00  (-0·01 – 0·00) | 0.23 |
| Sex  (M) | -0·19  (-0·27 – -0·11) | **<0·001** |
| EAT_3_ score ^ǂ^ | 0·00  (-0·04 – 0·05) | 0·83 |
| BW-PGS * Duration BF | -0·00  (-0·01 – 0·01) | 0·96 |

^§^ BW-PGS = birth weight polygenic score (standardised); ^†^ Duration of BF = Duration of any breastfeeding (mean-centred); ^‡^ EAT_1_ score (standardised) = quality of early life nutrition in first year of life; ^δ^ adjustment for population stratification; ^ǂ^ EAT_3_ score (standardised) = quality of early life nutrition in third year of life; ^ε^ Model 1 examines the effect of duration of breastfeeding and EAT_1_ score; ^φ^ Model 2 examine the effect of duration of breastfeeding and EAT_3_ score; All models adjusting for BW-PGS, sex and the first two principal components [PCs] (estimates not presented)

Supplementary Table 13. Association analyses for Plasma Glucose_F_ (Log Transformed)

| Percent Optimal Birth Weight (POBW) | Model 1 ^α^ | | Model 2 ^β^ | |
| --- | --- | --- | --- | --- |
| Predictors | Estimate  (95% CI) | P | Estimate  (95% CI) | P |
| Intercept | 1·59  (1·56 – 1·62) | **<0·0001** | 1·59  (1·56 – 1·62) | **<0·0001** |
| POBW ^γ^ | -0·01  (-0·01 – -0·000) | **0·030** | -0·01  (-0·01 – -0·00) | **0·022** |
| Duration BF  (months) ^†^ | -0·00  (-0·00 – 0·00) | 0·064 | -0·00  (-0·00 – -0·00) | **0·048** |
| Sex  (M) | 0·05  (0·04 – 0·06) | **<0·0001** | 0·05  (0·04 – 0·06) | **<0·0001** |
| EAT_1_ score ^‡^ | -0·00  (-0·00 – 0·00) | 0·36 | -0·00  (-0·00 – 0·00) | 0·40 |
| POBW * Duration BF |  |  | 0·00  (-0·00 – 0·00) | 0·17 |
| Birthweight Polygenic Score  (BW-PGS) | Model 3 ^ε^ | | Model 4 ^φ^ | |
| Predictors | Estimate  (95% CI) | P | Estimate  (95% CI) | P |
| Intercept | 1·59  (1·56 – 1·62) | **<0·0001** | 1·59  (1·56 – 1·62) | **<0·0001** |
| BW-PGS ^§^ | 0·00  (-0·00 – 0·01) | 0·43 | 0·00  (-0·00 – 0·01) | 0·40 |
| Duration BF  (months) ^†^ | -0·00  (-0·00 – -0·00) | **0·043** | -0·00  (-0·00 – -0·00) | **0·044** |
| Sex  (M) | 0·05  (0·04 – 0·06) | **<0·0001** | 0·05  (0·04 – 0·06) | **<0·0001** |
| EAT_1_ score ^‡^ | -0·00  (-0·00 – 0·00) | 0·43 | -0·00  (-0·00 – 0·00) | 0·44 |
| BW-PGS * Duration BF |  |  | -0·00  (-0·00 – 0·00) | 0·72 |

^γ^ POBW = Percent Optimal Birth Weight (standardised)^†^ Duration of BF = Duration of any breastfeeding (mean-centred); ^‡^ EAT_1_ score = quality of early life nutrition in first year of life; ^§^ BW-PGS = birth weight polygenic score (standardised); ^δ^ adjustment for population stratification; ^α^ Model 1 examines the effect of duration of breastfeeding adjusting for POBW, sex and EAT_1_ score; ^β^ Model 2 examines the effect modification of duration of breastfeeding by POBW adjusting for sex and EAT_1_ score; ^ε^ Model 3 examines the effect of duration of breastfeeding adjusting for BW-PGS, sex, EAT_1_ score and the first two principal components [PCs] (estimates not presented); ^φ^ Model 4 examines the effect modification of duration of breastfeeding by BW-PGS adjusting for sex, EAT_1_ score and the first two principal components [PCs] (estimates not presented)

Supplementary Table 14. Association analyses for Homeostatic Model Assessment for Insulin Resistance_F_ (HOMA-IR_F_) (Log Transformed)

| Percent Optimal Birth Weight (POBW) | Model 1 ^α^ | | Model 2 ^β^ | |
| --- | --- | --- | --- | --- |
| Predictors | Estimate  (95% CI) | P | Estimate  (95% CI) | P |
| Intercept | 0·76  (0·57 – 0·94) | **<0·0001** | 0·76  (0·58 – 0·95) | **<0·0001** |
| POBW ^γ^ | -0·06  (-0·10 – -0·02) | **0·0064** | -0·06  (-0·10 – -0·02) | **0·0075** |
| Duration BF  (months) ^†^ | -0·00  (-0·01 – 0·00) | 0·16 | -0·00  (-0·01 – 0·00) | 0·17 |
| Sex  (M) | -0·14  (-0·22 – -0·06) | **0.00050** | -0·14  (-0·22 – -0·06) | **0.00046** |
| EAT_1_ score ^‡^ | -0·00  (-0·01 – -0·00) | **0·031** | -0·01  (-0·01 – -0·00) | **0·030** |
| POBW * Duration BF |  |  | -0·00  (-0·01 – 0·00) | 0·67 |
| Birthweight Polygenic Score  (BW-PGS) | Model 3 ^ε^ | | Model 4 ^φ^ | |
| Predictors | Estimate  (95% CI) | P | Estimate  (95% CI) | P |
| Intercept | 0·73  (0·54 – 0·92) | **<0·0001** | 0·73  (0·55 – 0·92) | **<0·0001** |
| BW-PGS ^§^ | -0·01  (-0·05 – 0·03) | 0·55 | -0·01  (-0·05 – 0·03) | 0·54 |
| Duration BF  (months) ^†^ | -0·01  (-0·01 – 0·00) | 0·084 | -0·01  (-0·01 – 0·00) | 0·084 |
| Sex  (M) | -0·14  (-0·22 – -0·06) | **0·00068** | -0·14  (-0·22 – -0·06) | **0·00067** |
| EAT_1_ score ^‡^ | -0·00  (-0·01 – 0·00) | 0·065 | -0·00  (-0·01 – 0·00) | 0·064 |
| BW-PGS * Duration BF |  |  | 0·00  (-0·01 – 0·01) | 0·86 |

^γ^ POBW = Percent Optimal Birth Weight (standardised)^†^ Duration of BF = Duration of any breastfeeding (mean-centred); ^‡^ EAT_1_ score = quality of early life nutrition in first year of life; ^§^ BW-PGS = birth weight polygenic score (standardised); ^δ^ adjustment for population stratification; ^α^ Model 1 examines the effect of duration of breastfeeding adjusting for POBW, sex and EAT_1_ score; ^β^ Model 2 examines the effect modification of duration of breastfeeding by POBW adjusting for sex and EAT_1_ score; ^ε^ Model 3 examines the effect of duration of breastfeeding adjusting for BW-PGS, sex, EAT_1_ score and the first two principal components [PCs] (estimates not presented); ^φ^ Model 4 examines the effect modification of duration of breastfeeding by BW-PGS adjusting for sex, EAT_1_ score and the first two principal components [PCs] (estimates not presented)

Supplementary Table 15. Association Analyses for Fasting Low-Density-Lipoprotein-Cholesterol (mmol/L)

|  | Model 1 ^ε^ | |
| --- | --- | --- |
| Predictors | Estimate  (95% CI) | P |
| Intercept | 2·75  (2·67 – 2·83) | **<0·001** |
| BW-PGS ^§^ | 0·02  (-0·04 – 0·08) | 0·52 |
| Duration BF  (months) ^†^ | 0·00  (-0·01 – 0·01) | 0.61 |
| Sex  (M) | 0·02  (-0·09 – 0·13) | 0·71 |
| EAT_1_ score ^‡^ | -0·09  (-0·15 – -0·03) | **0·0020** |
| BW-PGS * Duration BF | -0·01  (-0·01 – 0·00) | 0·20 |
|  | Model 2 ^φ^ | |
| Predictors | Estimate  (95% CI) | P |
| Intercept | 2·74  (2·65 – 2·83) | **<0·001** |
| BW-PGS ^§^ | 0·04  (-0·03 – 0·10) | 0·24 |
| Duration BF  (months) ^†^ | 0·00  (-0·01 – 0·01) | 0.72 |
| Sex  (M) | 0·02  (-0·10 – 0·14) | 0·75 |
| EAT_3_ score ^ǂ^ | -0·05  (-0·11 – 0·01) | 0·080 |
| BW-PGS * Duration BF | -0·01  (-0·02 – -0·00) | **0·048** |

^§^ BW-PGS = birth weight polygenic score (standardised); ^†^ Duration of BF = Duration of any breastfeeding (mean-centred); ^‡^ EAT_1_ score (standardised) = quality of early life nutrition in first year of life; ^δ^ adjustment for population stratification; ^ǂ^ EAT_3_ score (standardised) = quality of early life nutrition in third year of life; ^ε^ Model 1 examines the effect of duration of breastfeeding and EAT_1_ score; ^φ^ Model 2 examines the effect of duration of breastfeeding and EAT_3_ score; All models adjusting for BW-PGS, sex and the first two principal components [PCs] (estimates not presented)

Supplementary Table 16. Association analyses for Total Cholesterol_F_ (mmol/L)

| Percent Optimal Birth Weight (POBW) | Model 1 ^α^ | | Model 2 ^β^ | |
| --- | --- | --- | --- | --- |
| Predictors | Estimate  (95% CI) | P | Estimate  (95% CI) | P |
| Intercept | 5·11  (4·80 – 5·41) | **<0·0001** | 5·10  (4·79 – 5·40) | **<0·0001** |
| POBW ^γ^ | -0·06  (-0·13 – 0·00) | 0·061 | -0·07  (-0·13 – 0·00) | **0·050** |
| Duration BF  (months) ^†^ | 0·01  (-0·00 – 0·02) | 0·15 | 0·01  (-0·00 – 0·02) | 0·18 |
| Sex  (M) | -0·21  (-0·34 – -0·08) | **0·0017** | -0·20  (-0·33 – -0·07) | **0·0028** |
| EAT_1_ score ^‡^ | -0·01  (-0·02 – -0·00) | **0·011** | -0·01  (-0·02 – -0·00) | **0·013** |
| POBW * Duration BF |  |  | 0·00  (-0·00 – 0·01) | 0·33 |
| Birthweight Polygenic Score  (BW-PGS) | Model 3 ^ε^ | | Model 4 ^φ^ | |
| Predictors | Estimate  (95% CI) | P | Estimate  (95% CI) | P |
| Intercept | 5·08  (4·78 – 5·39) | **<0·0001** | 5·08  (4·77 – 5·38) | **<0·0001** |
| BW-PGS ^§^ | -0·01  (-0·07 – 0·06) | 0·89 | -0·00  (-0·07 – 0·07) | 0·97 |
| Duration BF  (months) ^†^ | 0·01  (-0·00 – 0·02) | 0·24 | 0·01  (-0·00 – 0·02) | 0·23 |
| Sex  (M) | -0·21  (-0·34 – -0·08) | **0·0017** | -0·21  (-0·34 – -0·08) | **0·0020** |
| EAT_1_ score ^‡^ | -0·01  (-0·02 – -0·00) | **0·017** | -0·01  (-0·02 – -0·00) | **0·019** |
| BW-PGS * Duration BF |  |  | -0·00  (-0·01 – 0·01) | 0·51 |

^γ^ POBW = Percent Optimal Birth Weight (standardised)^†^ Duration of BF = Duration of any breastfeeding (mean-centred); ^‡^ EAT_1_ score = quality of early life nutrition in first year of life; ^§^ BW-PGS = birth weight polygenic score (standardised); ^δ^ adjustment for population stratification; ^α^ Model 1 examines the effect of duration of breastfeeding adjusting for POBW, sex and EAT_1_ score; ^β^ Model 2 examines the effect modification of duration of breastfeeding by POBW adjusting for sex and EAT_1_ score; ^ε^ Model 3 examines the effect of duration of breastfeeding adjusting for BW-PGS, sex, EAT_1_ score and the first two principal components [PCs] (estimates not presented); ^φ^ Model 4 examines the effect modification of duration of breastfeeding by BW-PGS adjusting for sex, EAT_1_ score and the first two principal components [PCs] (estimates not presented)

Supplementary Table 17. Association analyses for Triglycerides_F_ (Log Transformed)

| Percent Optimal Birth Weight (POBW) | Model 1 ^α^ | | Model 2 ^β^ | |
| --- | --- | --- | --- | --- |
| Predictors | Estimate  (95% CI) | P | Estimate  (95% CI) | P |
| Intercept | 0·04  (-0·10 – 0·19) | 0·57 | 0·05  (-0·10 – 0·20) | 0·52 |
| POBW ^γ^ | -0·04  (-0·07 – -0·01) | **0·023** | -0·04  (-0·07 – -0·00) | **0·030** |
| Duration BF  (months) ^†^ | 0·00  (-0·00 – 0·01) | 0·80 | 0·00  (-0·00 – 0·01) | 0·72 |
| Sex  (M) | 0·04  (-0·02 – 0·11) | 0·17 | 0·04  (-0·02 – 0·10) | 0·22 |
| EAT_1_ score ^‡^ | -0·00  (-0·00 – 0·00) | 0·47 | -0·00  (-0·00 – 0·00) | 0·44 |
| POBW * Duration BF |  |  | -0·00  (-0·01 – 0·00) | 0·34 |
| Birthweight Polygenic Score  (BW-PGS) | Model 3 ^ε^ | | Model 4 ^φ^ | |
| Predictors | Estimate  (95% CI) | P | Estimate  (95% CI) | P |
| Intercept | 0·03  (-0·12 – 0·17) | 0·74 | 0·03  (-0·12 – 0·18) | 0·71 |
| BW-PGS ^§^ | -0·00  (-0·03 – 0·03) | 0·93 | -0·00  (-0·04 – 0·03) | 0·86 |
| Duration BF  (months) ^†^ | 0·00  (-0·00 – 0·00) | 0·98 | 0·00  (-0·00 – 0·00) | 0·99 |
| Sex  (M) | 0·05  (-0·02 – 0·11) | 0·15 | 0·05  (-0·02 – 0·11) | 0·16 |
| EAT_1_ score ^‡^ | -0·00  (-0·00 – 0·00) | 0·62 | -0·00  (-0·00 – 0·00) | 0·60 |
| BW-PGS * Duration BF |  |  | 0·00  (-0·00 – 0·01) | 0·58 |

^γ^ POBW = Percent Optimal Birth Weight (standardised)^†^ Duration of BF = Duration of any breastfeeding (mean-centred); ^‡^ EAT_1_ score = quality of early life nutrition in first year of life; ^§^ BW-PGS = birth weight polygenic score (standardised); ^δ^ adjustment for population stratification; ^α^ Model 1 examines the effect of duration of breastfeeding adjusting for POBW, sex and EAT_1_ score; ^β^ Model 2 examines the effect modification of duration of breastfeeding by POBW adjusting for sex and EAT_1_ score; ^ε^ Model 3 examines the effect of duration of breastfeeding adjusting for BW-PGS, sex, EAT_1_ score and the first two principal components [PCs] (estimates not presented); ^φ^ Model 4 examines the effect modification of duration of breastfeeding by BW-PGS adjusting for sex, EAT_1_ score and the first two principal components [PCs] (estimates not presented)

Supplementary Table 18. Association analyses for High Density Lipoprotein Cholesterol_F_ (HDL-C_F_) (mmol/L)

| Percent Optimal Birth Weight (POBW) | Model 1 ^α^ | | Model 2 ^β^ | |
| --- | --- | --- | --- | --- |
| Predictors | Estimate  (95% CI) | P | Estimate  (95% CI) | P |
| Intercept | 0·32  (0·24 – 0·40) | **<0·0001** | 0·32  (0·24 – 0·40) | **<0·0001** |
| POBW ^γ^ | -0·02  (-0·03 – 0·00) | 0·086 | -0·02  (-0·03 – 0·00) | 0·093 |
| Duration BF  (months) ^†^ | 0·00  (0·00 – 0·01) | **0·0098** | 0·00  (0·00 – 0·01) | **0·0093** |
| Sex  (M) | -0·17  (-0·20 – -0·13) | **<0·0001** | -0·17  (-0·21 – -0·13) | **<0·0001** |
| EAT_1_ score ^‡^ | 0·00  (-0·00 – 0·00) | 0·26 | 0·00  (-0·00 – 0·00) | 0·27 |
| POBW * Duration BF |  |  | -0·00  (-0·00 – 0·00) | 0·73 |
| Birthweight Polygenic Score  (BW-PGS) | Model 3 ^ε^ | | Model 4 ^φ^ | |
| Predictors | Estimate  (95% CI) | P | Estimate  (95% CI) | P |
| Intercept | 0·32  (0·23 – 0·40) | **<0·0001** | 0·32  (0·24 – 0·40) | **<0·0001** |
| BW-PGS ^§^ | -0·01  (-0·03 – 0·01) | 0·15 | -0·02  (-0·03 – 0·00) | 0·11 |
| Duration BF  (months) ^†^ | 0·00  (0·00 – 0·01) | **0·022** | 0·00  (0·00 – 0·01) | **0·024** |
| Sex  (M) | -0·17  (-0·20 – -0·13) | **<0·0001** | -0·17  (-0·21 – -0·14) | **<0·0001** |
| EAT_1_ score ^‡^ | 0·00  (-0·00 – 0·00) | 0·20 | 0·00  (-0·00 – 0·00) | 0·22 |
| BW-PGS * Duration BF |  |  | 0·00  (-0·00 – 0·00) | 0·22 |

^γ^ POBW = Percent Optimal Birth Weight (standardised)^†^ Duration of BF = Duration of any breastfeeding (mean-centred); ^‡^ EAT_1_ score = quality of early life nutrition in first year of life; ^§^ BW-PGS = birth weight polygenic score (standardised); ^δ^ adjustment for population stratification; ^α^ Model 1 examines the effect of duration of breastfeeding adjusting for POBW, sex and EAT_1_ score; ^β^ Model 2 examines the effect modification of duration of breastfeeding by POBW adjusting for sex and EAT_1_ score; ^ε^ Model 3 examines the effect of duration of breastfeeding adjusting for BW-PGS, sex, EAT_1_ score and the first two principal components [PCs] (estimates not presented); ^φ^ Model 4 examines the effect modification of duration of breastfeeding by BW-PGS adjusting for sex, EAT_1_ score and the first two principal components [PCs] (estimates not presented)

Supplementary Table 19. Three-way interaction association analysis to examine the effect of breastfeeding by POBW and sex for body mass index (kg/m^2^)

| Percent Optimal Birth Weight (POBW) |  | |
| --- | --- | --- |
| Predictors | Estimate  (95% CI) | P |
| Intercept | 27·82  (25·88 – 29·77) | **<0·0001** |
| POBW ^γ^ | 0·67  (0·05 – 1·28) | **0·033** |
| Duration BF  (months) ^†^ | -0·10  (-0·18 – -0·0) | **0·029** |
| Sex  (M) | 0·13  (-0·70 – 0·97) | 0·75 |
| EAT_1_ score ^‡^ | -0·06  (-0·10 – -0·02) | **0·0055** |
| POBW * Duration BF | 0·03  (-0·04 – 0·09) | 0·48 |
| POBW * Sex (M) | -0·50  (-1·35 – 0·35) | 0·25 |
| Duration BF * Sex (M) | 0·05  (-0·07 – 0·17) | 0·45 |
| POBW * Duration BF * Sex (M) | -0·11  (-0·21 – 0·00) | 0·053 |

^γ^ POBW = Percent Optimal Birth Weight (standardised)^†^ Duration of BF = Duration of any breastfeeding (mean-centred); ^‡^ EAT_1_ score = quality of early life nutrition in first year of life

Supplementary Table 20. Three-way interaction association analysis to examine the effect modification of duration of breastfeeding by POBW and sex on systolic blood pressure (mmHg)

| Percent Optimal Birth Weight (POBW) |  | |
| --- | --- | --- |
| Predictors | Estimate  (95% CI) | P |
| Intercept | 118·23  (114·53 – 121·94) | **<0·0001** |
| POBW ^§^ | -0·65  (-1·82 – 0·53) | 0·28 |
| Duration BF  (months) ^†^ | 0·06  (-0·10 – 0·23) | 0·46 |
| Sex  (M) | 9·52  (7·91 – 11·12) | **<0·0001** |
| EAT_1_ score ^‡^ | -0·11  (-0·19 – -0·02) | **0·012** |
| POBW * Duration BF | 0·07  (-0·07 – 0·21) | 0·31 |
| POBW * Sex (M) | -0·06  (-1·70 – 1·58) | 0·94 |
| Duration BF * Sex (M) | 0·08  (-0·15 – 0·31) | 0·51 |
| POBW * Duration BF * Sex (M) | -0·136  (-0·36 – 0·05) | 0·14 |

^γ^ POBW = Percent Optimal Birth Weight (standardised)^†^ Duration of BF = Duration of any breastfeeding (mean-centred); ^‡^ EAT_1_ score = quality of early life nutrition in first year of life

Supplementary Table 21. Three-way interaction association analysis to examine the effect modification of duration of breastfeeding by BW-PGS and sex for body mass index (kg/m^2^)

| Birthweight Polygenic Score (BW-PGS) |  | |
| --- | --- | --- |
| Predictors | Estimate  (95% CI) | P |
| Intercept | 27·55  (25·60 – 29·50) | **<0·0001** |
| BW-PGS ^§^ | 0·25  (-0·36 – 0·87) | 0·42 |
| Duration BF  (months) ^†^ | -0·07  (-0·15 – 0·01) | 0·087 |
| Sex  (M) | 0·21  (-0·62 – 1·04) | 0·62 |
| EAT_1_ score ^‡^ | -0·06  (-0·10 – -0·01) | **0·011** |
| BW-PGS * Duration BF | -0·09  (-0·16 – -0·02) | **0·0095** |
| BW-PGS * Sex (M) | -0·17  (-1·03 – 0·69) | 0·70 |
| Duration BF * Sex (M) | 0·04  (-0·08 – 0·15) | 0·56 |
| BW-PGS * Duration BF * Sex (M) | 0·08  (-0·03 – 0·20) | 0·14 |

^§^ BW-PGS = birth weight polygenic score (standardised);^†^ Duration of BF = Duration of any breastfeeding (mean-centred); ^‡^ EAT_1_ score = quality of early life nutrition in first year of life; ^δ^ adjustment for the first two principal components [PCs] (estimates not presented)

Supplementary Table 22. Three-way interaction association analysis to examine the effect modification of duration of breastfeeding by BW-PGS and sex on systolic blood pressure (mmHg)

| Birthweight Polygenic Score (BW-PGS) |  | |
| --- | --- | --- |
| Predictors | Estimate  (95% CI) | P |
| Intercept | 118·14  (114·43 – 121·86) | **<0·0001** |
| BW-PGS ^§^ | 0·29  (-0·87 – 1·45) | 0·62 |
| Duration BF  (months) ^†^ | 0·06  (-0·09 – 0·21) | 0·43 |
| Sex  (M) | 9·48  (7·89 – 11·077) | **<0·0001** |
| EAT_1_ score ^‡^ | -0·10  (-0·18 – -0·02) | **0·016** |
| BW-PGS * Duration BF | -0·09  (-0·22 – 0·05) | 0·20 |
| BW-PGS * Sex (M) | -0·20  (-1·83 – 1·43) | 0·81 |
| Duration BF * Sex (M) | 0·14  (-0·08 – 0·36) | 0·22 |
| BW-PGS * Duration BF * Sex (M) | -0·09  (-0·30 – 0·13) | 0·42 |

^§^ BW-PGS = birth weight polygenic score (standardised);^†^ Duration of BF = Duration of any breastfeeding (mean-centred); ^‡^ EAT_1_ score = quality of early life nutrition in first year of life; ^δ^ adjustment for the first two principal components [PCs] (estimates not presented)

Supplementary Table 23. Association Analyses for Body Mass Index (BMI, kg/m^2^)

|  | Model 1 ^ε^ | |
| --- | --- | --- |
| Predictors | Estimate  (95% CI) | P |
| Intercept | 27·71  (25·76 – 29·66) | **<0·001** |
| BW-PGS ^§^ | 0·10  (-0·33 – 0·52) | 0·66 |
| Duration BF  (months) ^†^ | -0·06  (-0·14 – 0·02) | 0.14 |
| Sex  (M) | 0·17  (-0·66 – 1·00) | 0·69 |
| EAT_1_ score ^‡^ | -0·06  (-0·10 – -0·02) | **0·0080** |
| Duration BF * Sex (M) | 0·03  (-0·09 – 0·14) | 0·67 |

^§^ BW-PGS = birth weight polygenic score (standardised); ^†^ Duration of BF = Duration of any breastfeeding (mean-centred); ^‡^ EAT_1_ score (standardised) = quality of early life nutrition in first year of life; ^δ^ adjustment for population stratification; ^ǂ^ EAT_3_ score (standardised) = quality of early life nutrition in third year of life; ^ε^ Model 1 examines the effect of duration of breastfeeding and EAT_1_ score; All models adjusting for BW-PGS, sex and the first two principal components [PCs] (estimates not presented)

Supplementary Table 24. Association Analyses for Systolic Blood Pressure (SBP, mmHg)

|  | Model 1 ^ε^ | |
| --- | --- | --- |
| Predictors | Estimate  (95% CI) | P |
| Intercept | 118·28  (114·57 – 121·99) | **<0·001** |
| BW-PGS ^§^ | 0·05  (-0·77 – 0·86) | 0·91 |
| Duration BF (months) ^†^ | 0·07  (-0·09 – 0·20) | 0.38 |
| Sex  (M) | 9·39  (7·80 – 10·98) | **<0·001** |
| EAT_1_ score ^‡^ | -0·10  (-0·19 – -0·02) | **0·014** |
| Duration BF * Sex (M) | 0·10  (-0·12 – 0·32) | 0·81 |

^§^ BW-PGS = birth weight polygenic score (standardised); ^†^ Duration of BF = Duration of any breastfeeding (mean-centred); ^‡^ EAT_1_ score (standardised) = quality of early life nutrition in first year of life; ^δ^ adjustment for population stratification; ^ǂ^ EAT_3_ score (standardised) = quality of early life nutrition in third year of life; ^ε^ Model 1 examines the effect of duration of breastfeeding and EAT_1_ score; All models adjusting for BW-PGS, sex and the first two principal components [PCs] (estimates not presented)

**Supplementary Figures**

| **Supplementary Figure 1** | Marginal effect of the duration of any breastfeeding on:   1. BMI across the POBW (standardised) spectrum 2. BMI across the BW-PGS (standardised) spectrum 3. SBP across the POBW (standardised) spectrum 4. SBP across the BW-PGS (standardised) spectrum |
| --- | --- |
| **Supplementary Figure 2** | The relationship between duration of any breastfeeding (by POBW) and health measures at 22 years of age:   1. DEXA measured body fat percentage 2. Diastolic BP (mmHg) 3. Fasting Plasma Glucose (mmol/L) 4. Fasting HOMA-IR 5. Fasting Total Cholesterol (mmol/L) 6. Fasting Triglycerides (mmol/L) 7. Fasting HDL-c (mmol/L) |
| **Supplementary Figure 3** | The relationship between duration of any breastfeeding (by BW-PGS) and health measures at 22 years of age:   1. DEXA measured body fat percentage 2. Diastolic BP (mmHg) 3. Fasting Plasma Glucose (mmol/L) 4. Fasting HOMA-IR 5. Fasting Total Cholesterol (mmol/L) 6. Fasting Triglycerides (mmol/L) 7. Fasting HDL-c (mmol/L) |


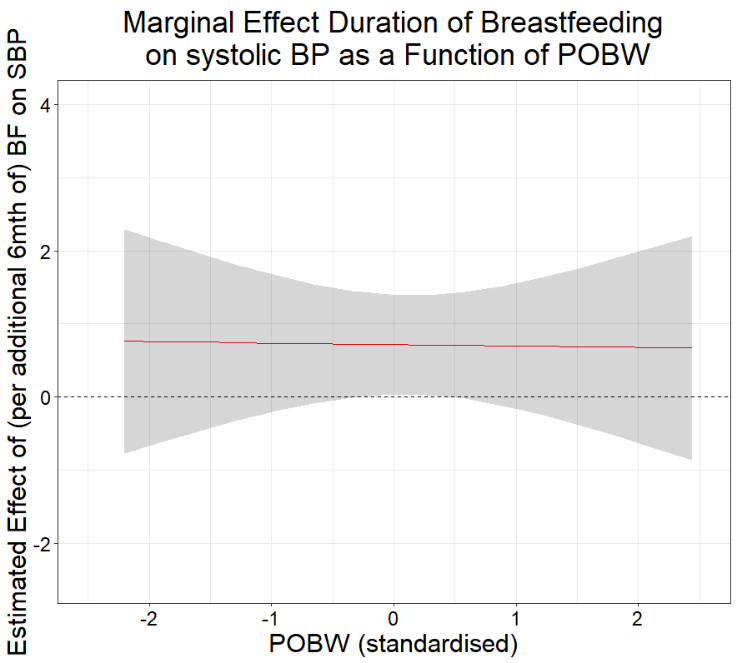

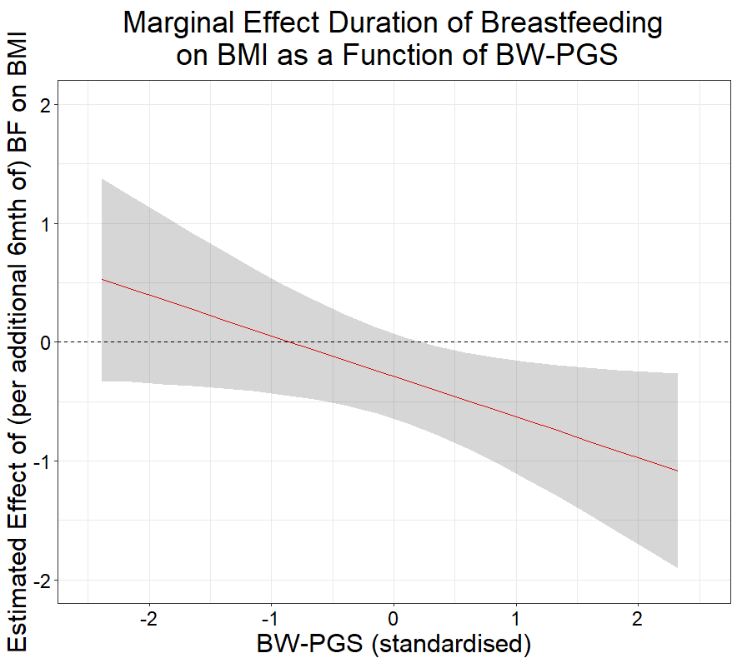

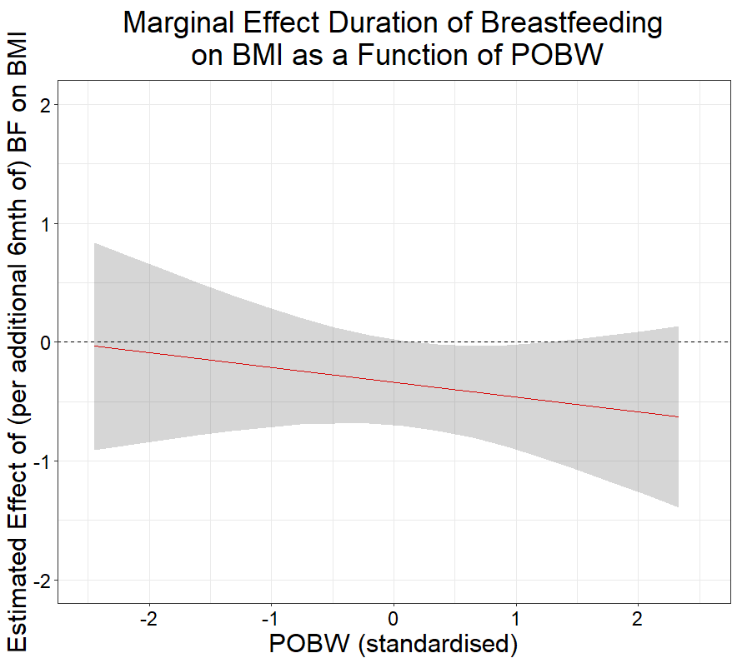


A

B

C

D


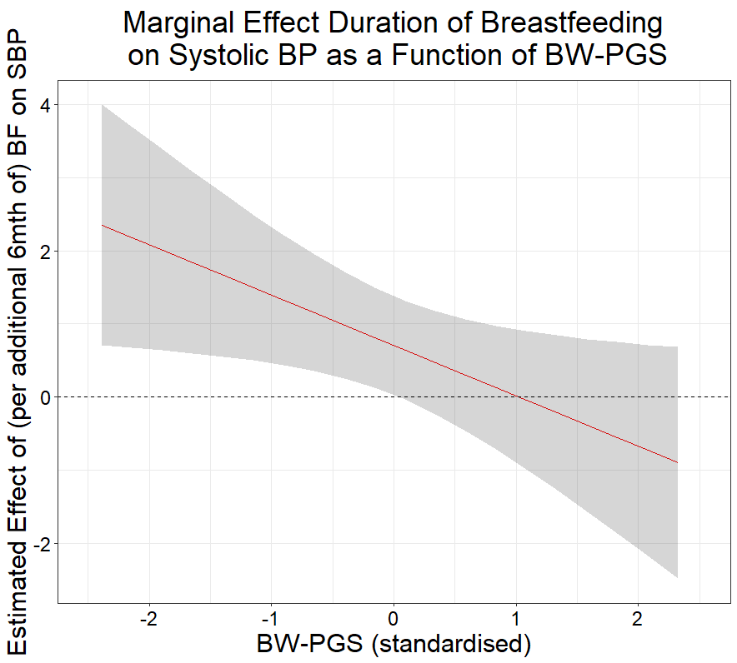


Supplementary Figure 1. Marginal effect of duration of breastfeeding on BMI and systolic BP at 22 years of age across the POBW and BW-PGS spectrum. The effect of breastfeeding on the outcome is not significant different to zero where the confidence interval crosses the horizontal line of zero. (A): Marginal effect of duration of breastfeeding on BMI at 22 years of age across the POBW (standardised) spectrum adjusting for sex and diet quality in the first year of life (EAT_1_); (B): Marginal effect of duration of breastfeeding on systolic BP at 22 years of age across the POBW (standardised) spectrum adjusting for sex and diet quality in the first year of life (EAT_1_); (C): Marginal effect of duration of breastfeeding on BMI at 22 years of age across the BW-PGS (standardised) spectrum adjusting for sex, diet quality in the first year of life (EAT_1_) and population stratification (PC1 and PC2); (D): Marginal effect of duration of breastfeeding on systolic BP at 22 years of age across the BW-PGRS (standardised) spectrum adjusting for sex, diet quality in the first year of life (EAT_1_) and population stratification (PC1 and PC2) (Figure generated using ggplot2 in R^1,2^)

Supplementary Figure 2. The relationship between duration of any breastfeeding and cardiovascular risk factor s in four areas of health outcomes for POBW that is one standard deviation above the mean, and for POBW that is one standard deviation below the mean; all models are adjusted for sex and diet quality in the first year of life (EAT_1_). _F_ denotes fasting. (A): DEXA measured Body Fait Percentage at 20 years of age; (B): Diastolic blood pressure (DBP) at 22 years of age; (C): Fasting plasma glucose level at 22 years of age; and (D): Fasting HOMA-IR level at 22 years of age; (E): Fasting total cholesterol level at 22 years of age; (F): Fasting triglycerides level at 22 years of age; (G): Fasting high density lipoprotein (HDL-c) level at 22 years of age (Figure generated using ggplot2 in R^1,2^)


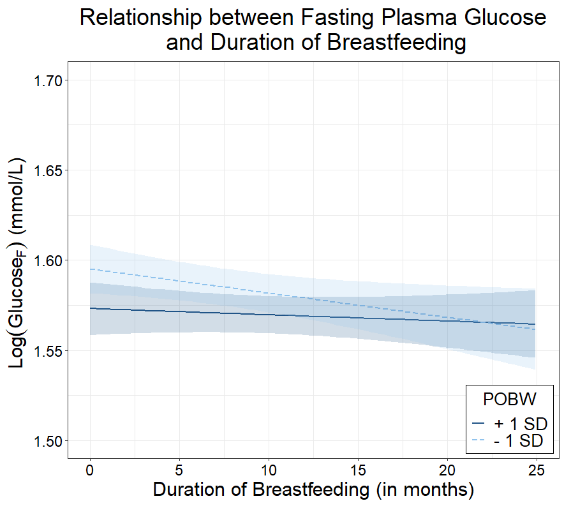

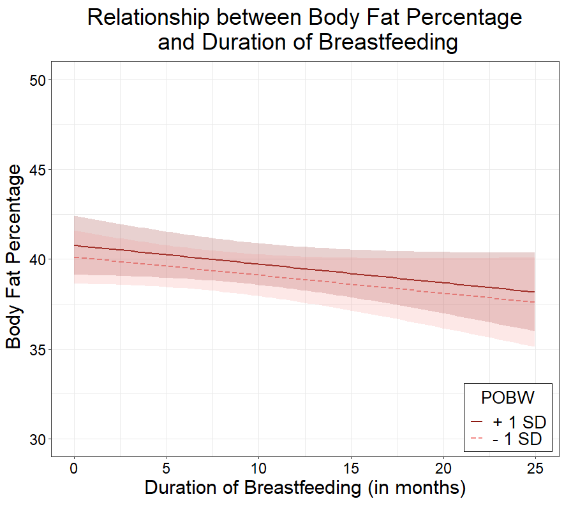

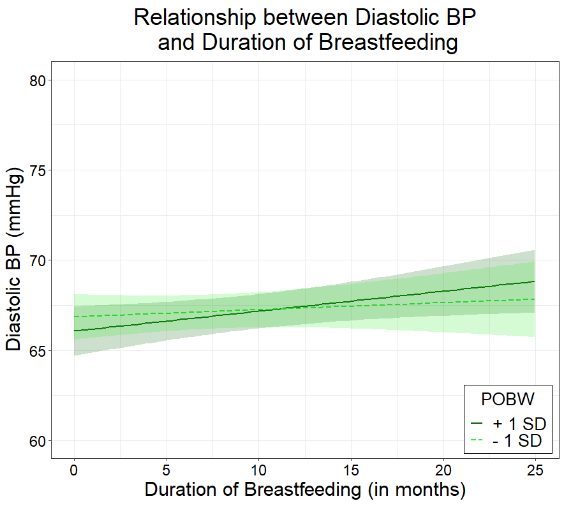

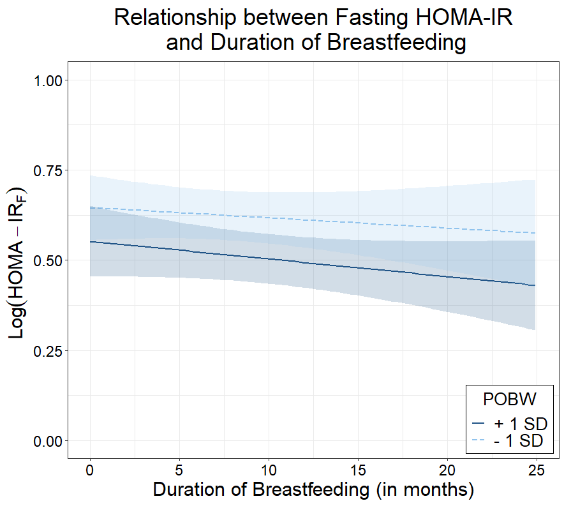

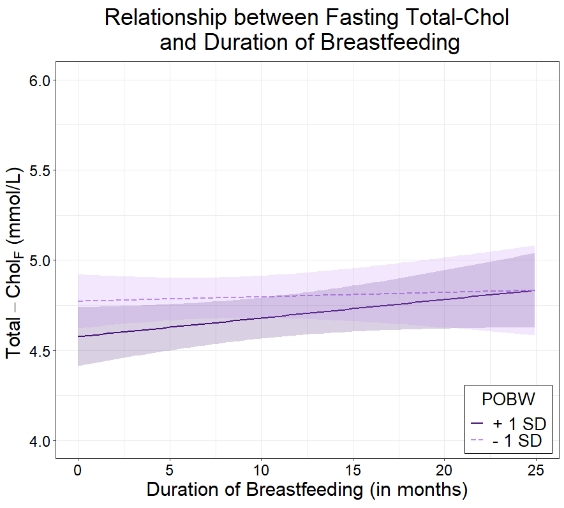

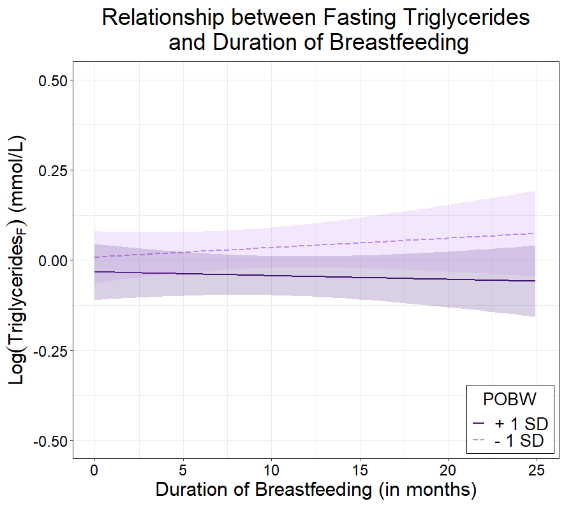

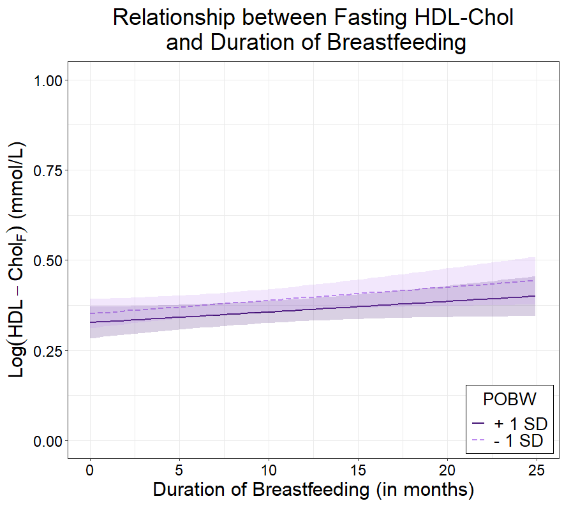


A

B

C

D

E

F

G

Supplementary Figure 3. The relationship between duration of any breastfeeding and cardiovascular risk factors in four areas of health outcomes for BW-PGS that is one standard deviation above the mean, and for BW-PGS that is one standard deviation below the mean all models are adjusted for sex, diet quality in the first year of life (EAT_1_), and population stratification (PC1 and PC2). _F_ denotes fasting. (A): DEXA measured Body Fait Percentage at 20 years of age; (B): Diastolic blood pressure (DBP) at 22 years of age; (C): Fasting plasma glucose level at 22 years of age; and (D): Fasting HOMA-IR level at 22 years of age; (E): Fasting total cholesterol level at 22 years of age; (F): Fasting triglycerides level at 22 years of age; (G): Fasting high density lipoprotein (HDL-c) level at 22 years of age (Figure generated using ggplot2 in R^1,2^)


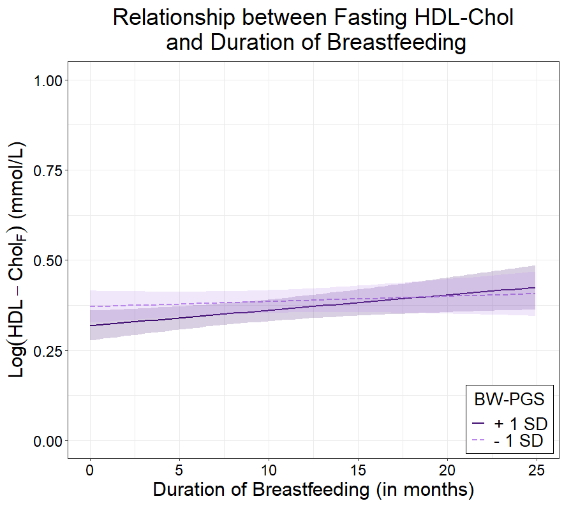

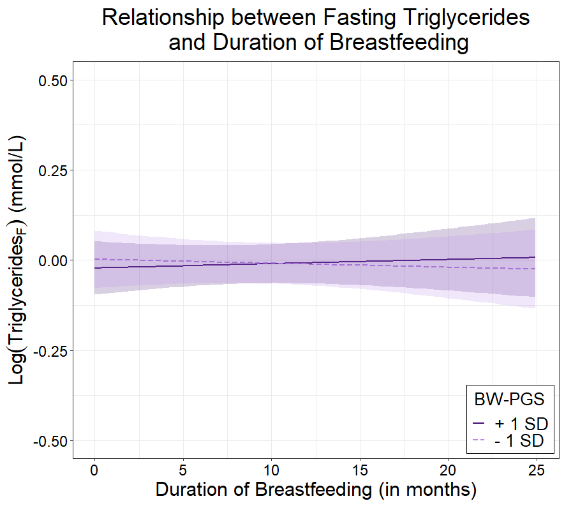

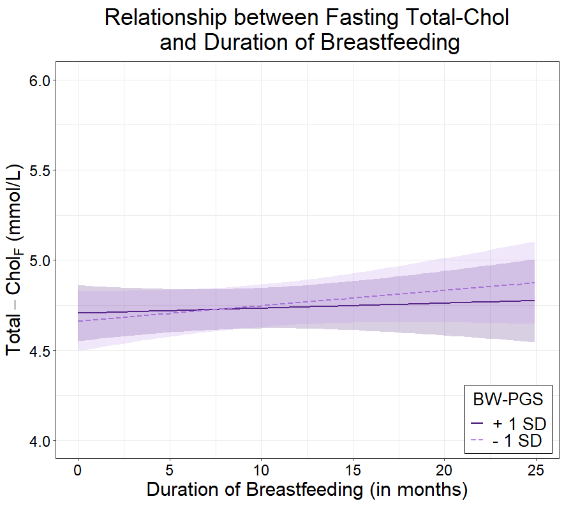

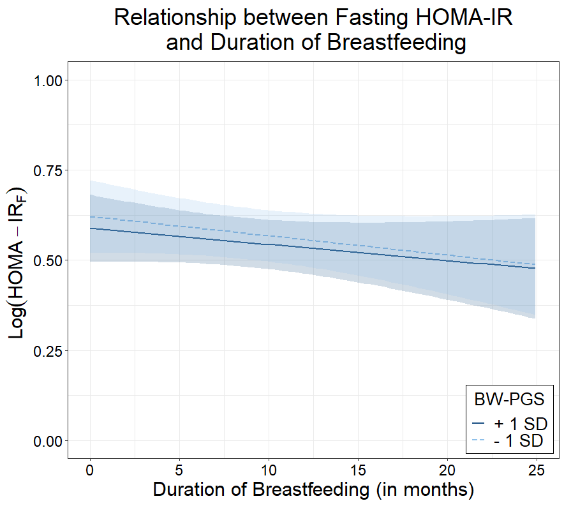

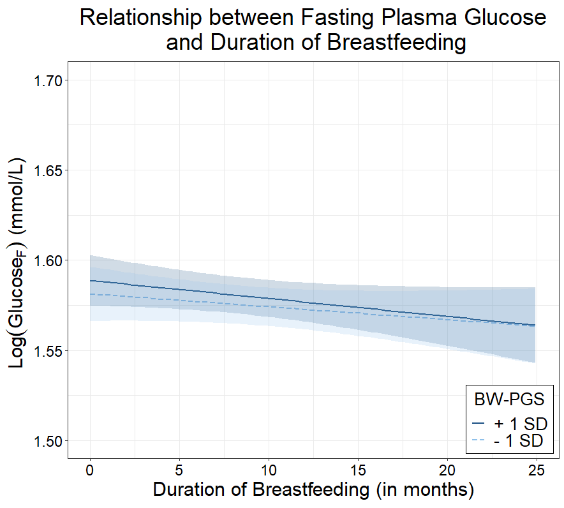

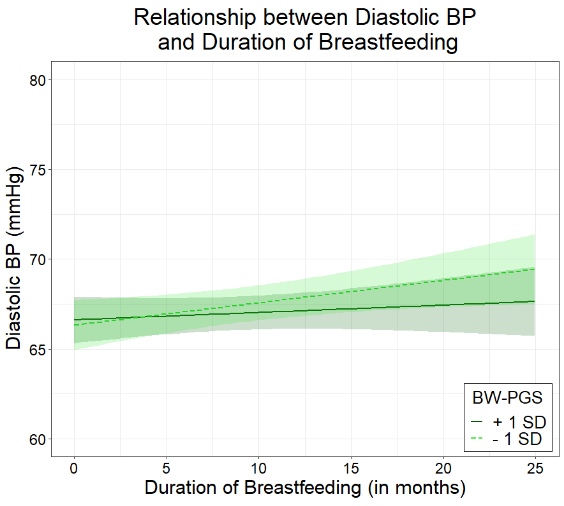

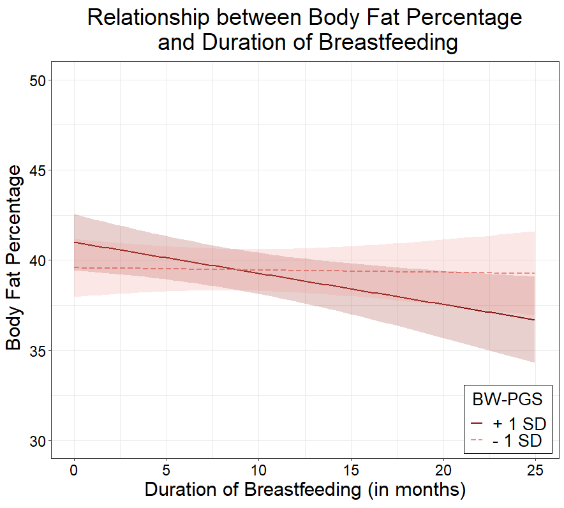


A

B

C

D

E

F

G

**References**

1. Wickham H. ggplot2: Elegant Graphics for Data Analysis: Springer-Verlag New York; 2016.

2. R Development Core Team. R: A language and Environment for Statistical Computing. R Foundation for Statistical Computing; 2019.
